# Cotargeting CHK1 and PI3K Synergistically Suppresses Tumor Growth of Oral Cavity Squamous Cell Carcinoma in Patient-Derived Xenografts

**DOI:** 10.3390/cancers12071726

**Published:** 2020-06-29

**Authors:** Chia-Yu Yang, Chiao-Rou Liu, Ian Yi-Feng Chang, Chun-Nan OuYang, Chia-Hsun Hsieh, Yen-Lin Huang, Chun-I Wang, Fei-Wen Jan, Wan-Ling Wang, Ting-Lin Tsai, Hsuan Liu, Ching-Ping Tseng, Yu-Sun Chang, Chih-Ching Wu, Kai-Ping Chang

**Affiliations:** 1 Department of Microbiology and Immunology, College of Medicine, Chang Gung University, Taoyuan 33302, Taiwan; chiayu-yang@mail.cgu.edu.tw (C.-Y.Y.); nancy97446@gmail.com (C.-R.L.); a39226480@yahoo.com.tw (F.-W.J.); sweet216014@hotmail.com (W.-L.W.); timmylife17@gmail.com (T.-L.T.); 2 Graduate Institute of Biomedical Sciences, College of Medicine, Chang Gung University, Taoyuan 33302, Taiwan; liu-hsuan@mail.cgu.edu.tw (H.L.); ysc@mail.cgu.edu.tw (Y.-S.C.); 3 Department of Otolaryngology Head and Neck Surgery, Chang Gung Memorial Hospital, Taoyuan 33305, Taiwan; yeewang0330@gmail.com; 4 Molecular Medicine Research Center, Chang Gung University, Taoyuan 33302, Taiwan; ian.yfchang@gmail.com (I.Y.-F.C.); oychunnan@gmail.com (C.-N.O.); 5 Department of Medical Biotechnology and Laboratory Science, College of Medicine, Chang Gung University, Taoyuan 33302, Taiwan; ctseng@mail.cgu.edu.tw; 6 Division of Hematology-Oncology, Department of Internal Medicine, Chang Gung Memorial Hospital, Taoyuan 33305, Taiwan; wisdom5000@gmail.com; 7 College of Medicine, Chang Gung University, Taoyuan 33302, Taiwan; 8 Department of Pathology, Chang Gung Memorial Hospital, Taoyuan 33305, Taiwan; louisyhuang@gmail.com; 9 Department of Cell and Molecular Biology, College of Medicine, Chang Gung University, Taoyuan 33302, Taiwan; 10 Division of Colon and Rectal Surgery, Chang Gung Memorial Hospital, Taoyuan 33305, Taiwan; 11 Department of Laboratory Medicine, Chang Gung Memorial Hospital, Taoyuan 33305, Taiwan

**Keywords:** oral cavity squamous cell carcinomas, CHK1, PI3K, patient-derived xenograft

## Abstract

Oral cavity squamous cell carcinomas (OSCCs) are aggressive tumors, and their recurrence leads to poor prognosis and reduced survival rates. This study aimed to identify therapeutic targets and to evaluate the efficacy of targeted inhibitors in OSCC patient-derived xenograft (PDX) models. Herein, we reported that OSCC PDXs recapitulated the genomic signatures of their paired primary tumors and the expression of *CHEK1*, *PIK3CA*, and *PIK3CD* was significantly upregulated in OSCC. The antitumor efficacy of CHK1 inhibitors (PF477736, AZD7762, LY2606368) and PI3K inhibitors (BYL719, GDC0941, GSK1059615) was investigated in OSCC cell lines and PDX models. Targeting either CHK1 or PI3K effectively inhibited cell proliferation and colony formation by inducing cell cycle arrest and apoptosis in in vitro cell-based assays. Cisplatin-based chemotherapy combined with CHK1 inhibitor treatment synergistically inhibited cell proliferation by suppressing CHK1 phosphorylation and inducing PARP cleavage. Furthermore, compared with monotherapy, cotreatment with CHK1 and PI3K inhibitors exerted synergistic anticancer effects by suppressing CHK1, AKT, and 4E-BP1 phosphorylation. In summary, our study identified CHK1 and PI3K as promising targets, especially in a dual treatment strategy combining a CHK1 inhibitor with cisplatin or a PI3K inhibitor as a novel therapeutic approach for OSCC patients with aberrant cell cycle regulation and PI3K signaling activation.

## 1. Introduction

Oral cavity squamous cell carcinoma (OSCC) is an aggressive tumor and is the most common malignancy of the oral cavity, accounting for up to 90% of all malignant neoplasms of the oral cavity [1,2,3]. The incidence of OSCC appears to be increasing worldwide, especially in Asia, and this common cancer is the fourth most prevalent cancer among males in Taiwan [4,5]. Several molecular alterations, including cell cycle dysregulation and EGFR pathway amplification, have been described as key players in OSCC pathogenesis [3,6,7,8]. Cigarette smoking, alcohol consumption, and betel nut chewing are potential risk factors that contribute to the high occurrence of oral cancer [2,9,10]. The current treatment for OSCC patients is surgical excision at an early stage, with subsequent chemotherapy or radiotherapy. Postoperative concurrent chemoradiotherapy is an acceptable standard therapy for patients at high risk of recurrence, as determined by pathological examination [11]. The overall relative 5-year survival rate of OSCC is approximately 60%; treatments for early-stage OSCC are relatively effective, but nearly 65% of patients present with advanced disease (stages III and IV), and only approximately 50% of these patients can be cured [12]. Cisplatin-based chemotherapy or cetuximab is currently a standard treatment for recurrent/metastatic OSCC [13]. However, not all patients respond effectively to EGFR-targeted therapy. The vast majority of OSCC patients are resistant to this therapy, indicating that other effective targeted therapies are needed for OSCC patients with different genetic and molecular signatures.

Dysregulation of the cell cycle, apoptosis, and/or DNA damage response pathways is a common cause for the development of cancers, including oral cancer [14]. Therefore, numerous inhibitors targeting these pathways have been evaluated as therapeutic agent candidates for oral cancer. For instance, palbociclib and abemaciclib, which are inhibitors of the cell cycle-related kinases CDK4 and CDK6, show an anti-tumor effect in a patient-derived xenograft (PDX) model of head and neck squamous cell carcinoma (HNSCC) [15]. Recent studies demonstrated that targeted therapy based on inhibiting the DNA damage response and p53 provides significant opportunities for HNSCC treatment [16,17]. In addition, inhibitors to pathways that involve phosphoinositide 3-kinase (PI3K), survivin, Janus kinase (JAK), matrix metallopeptidase 9 (MMP-9), vascular endothelial growth factor (VEGF), and mitogen-activated protein kinases (MAPKs) are also under investigation to determine their treatment efficacy in squamous cell carcinoma [14,18,19].

Cancer cell lines derived from tumor tissues have been widely used for the development of cancer treatment approaches. However, because of the genetic variations and lack of tumor microenvironment in vitro, cell lines may not be fully useful for establishment of treatment methods. PDX models are recognized as accurate and clinically relevant models and are used as powerful tools for characterizing tumor biology and screening drugs [15,20,21]. However, OSCC PDX models have not been reported in Asian populations. Furthermore, comprehensive characterization of genomic signatures in primary tissues and paired xenografts could provide valid evidence to support functional and therapeutic studies in cancer research. Herein, we established three PDX models of late-stage OSCC. We comprehensively analyzed the genomic and transcriptomic profiles of the paired primary tumors and xenografts to identify actionable therapeutic targets and determined the efficacy of potential small molecule inhibitors in OSCC cell lines, cell line-derived xenografts (CDXs), and PDXs. In the present study, we demonstrate that *CHEK1*, *PIK3CA*, and *PIK3CD* are significantly upregulated in OSCC and provide evidence that a dual treatment strategy that combine a CHK1 inhibitor with a PI3K inhibitor or cisplatin is a potential therapeutic option for patients.

## 2. Results

### 2.1. Establishment and Characterization of the PDX Models

**Figure 1:**
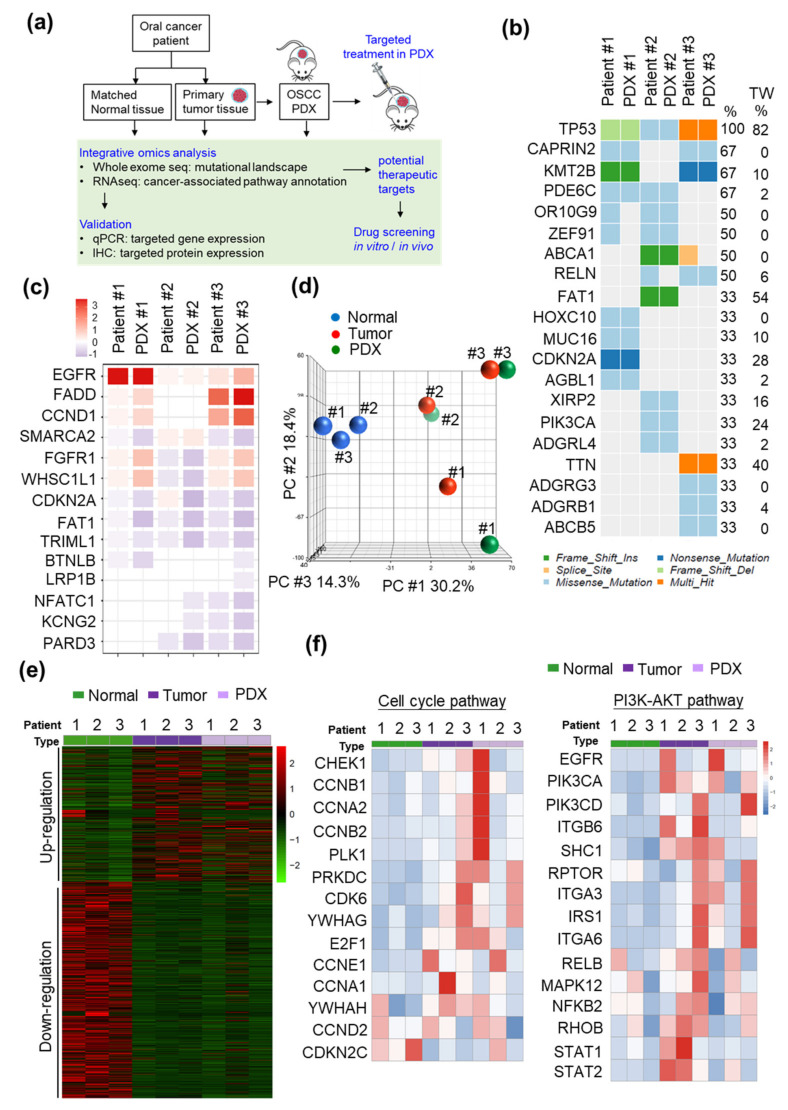
Integrated genomic and transcriptomic analysis of OSCC PDXs. (**a**) Workflow for the integrated genomic characterization and evaluation of the antitumor efficacy of potential inhibitors in OSCC. (**b**) A heatmap of the genomic mutational landscape of the three patients and paired PDXs was generated from the whole-exome sequencing data. The right column shows the mutation frequency of individual genes in the 50 OSCC—Taiwan samples. **(c)** Heatmap representation of the copy number variations in the patients and paired PDXs. (**d**) PCA of the transcriptome datasets for nine samples. (**e**) Heatmap of the differentially regulated genes (DEGs) in OSCC patients and PDXs demonstrating separation of the expression profiles of normal and tumor tissues. (**f**) Heat maps showing the differentially regulated genes involved in the cell cycle pathway (left panel) and PI3K-AKT pathway (right panel). The signaling intensities for genes are standardized for visualization, and the expression levels are Z-scored. Z-scores were calculated as Z = (X - μ_x_)/σ_x_, where X represents the individual raw expression value, μ_x_ represents the mean raw expression value for the genes across different sample types, and σ_x_ is the standard deviation associated with μ_x_.

To develop patient-tailored treatment strategies for OSCC, we established three PDX models with tumors from Stage IV OSCC patients (Table 1). To evaluate the tumor-associated biological pathways and seek potential druggable targets in OSCC, the mutational landscape and tumor transcriptome profiles of the tumors from these patients and the paired xenografts were determined by whole-exome sequencing and RNA sequencing (RNA-seq), respectively. The detailed workflow of our experiments is shown in Figure 1a. Whole-exome sequencing analysis (75-Mbp target region, mean depth in tumor tissue = 213.4 ± 20.6, and mean depth in normal tissue = 129.4 ± 5.3) revealed 1605 somatic single-nucleotide variants (SNVs). According to our previous report, the five most frequently mutated genes in OSCC patients were *TP53*, *FAT1*, *NOTCH*, *PIK3CA*, and *CDKN2A* [6]. Consistent with previous findings, Patient #1 harbored *TP53* and *CDKN2A* mutations; Patient #2 harbored *TP53*, *FAT1*, and *PIK3CA* mutations; and Patient #3 harbored a *TP53* mutation (Figure 1b). In addition, whole-exome sequencing analysis indicated that the copy number variation in individual patients was comparable to that in their corresponding xenografts, including in significantly amplified/deleted regions encompassing genes such as *EGFR*, *FADD*, *CCND1*, *CDKN2A*, and *FAT1* (Figure 1c).

We then analyzed the transcriptome profiles of these patients and the PDXs. Our RNA-seq analyses revealed 32,200 genes, and the mean read count was 36.5 ± 0.76 million. The genes with a 2-fold expression difference in tumor tissue compared with the adjacent normal tissue were considered differentially expressed genes (DEGs; Figure S1a). Principal component analysis (PCA) and hierarchical clustering were performed on the RNA-seq data to comparatively characterize the overall transcriptome profiles (Figure 1d,e). We then performed biological pathway annotation by gene set enrichment analysis (GSEA) to identify the pathogenesis-associated pathways that may contribute to tumorigenesis in individual patients. Pathway analysis of the upregulated genes via GSEA revealed significant enrichment of the differentially expressed genes in several pathways, such as the cell cycle, focal adhesion, and extracellular matrix (ECM)-receptor interaction (Figure S1b). The differentially expressed genes in individual patients were then subjected to the cancer hallmark enrichment analysis; cell cycle-related pathways and the PI3K-AKT pathway were significantly enriched in all three OSCC patients (*p* < 0.01; Figure S2). Additionally, these OSCC PDX models recapitulated genomic characteristics similar to those of oral cancer patients, in whom several genes involved in the cell cycle and PI3K-AKT pathways were dysregulated (Figure 1f). According to our integrated tumor and xenograft analysis, the overall expression patterns of key cancer-associated genes were recapitulated in patients and their paired PDXs. These results suggest that our newly established OSCC PDXs are relevant preclinical models.

### 2.2. CHEK1 and PIK3CA/PIK3CD are Overexpressed in OSCC

We cross referenced these data with our previously published OSCC transcriptome datasets containing 39 paired samples (uploaded to the NCBI Sequence Read Archive (SRA) database under accession code SRP078156). The upregulated genes (fold change ≥ 2; *p* < 0.05) in the 39 paired OSCC transcriptome datasets were analyzed with the DAVID and GSEA bioinformatics tools. The significantly enriched cancer-associated pathways included the cell cycle, PI3K-AKT signaling pathway, focal adhesion, and ECM-receptor interaction (Figure S3a,b). The significantly upregulated genes in OSCC involved in the cell cycle and PI3K-AKT pathways are shown in red (Figure S3c). These results indicate that the genetic signatures of our newly established PDXs reflect those of the majority of OSCC patients.

**Figure 2:**
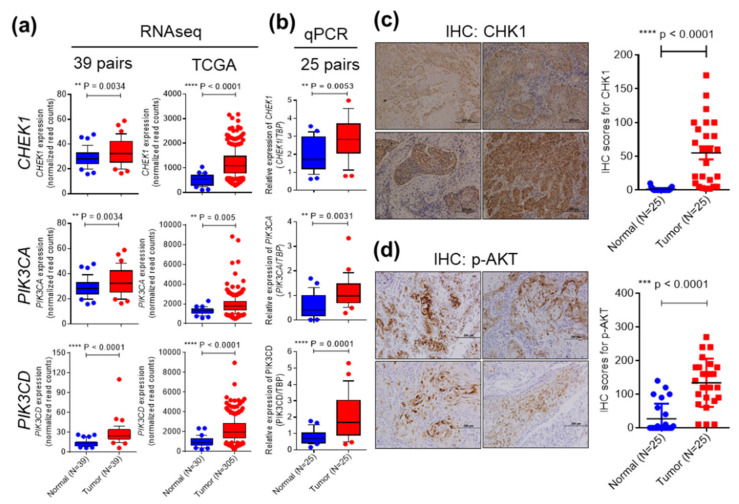
Expression of *CHEK1*, *PIK3CA*, and *PIK3CD* is significantly upregulated in OSCC. (**a**) Tissue transcriptome datasets of the dysregulated genes in 39 paired samples from Taiwanese OSCC patients and 305 OSCC patients from TCGA were analyzed. Upon read alignment, the gene expression levels of *CHEK1*, *PIK3CA*, and *PIK3CD* were determined based on the normalized read count values. (**b**) *CHEK1*, *PIK3CA*, and *PIK3CD* gene expression was analyzed by qRT-PCR in paired tumor tissues and adjacent normal tissues of 25 OSCC patients. The expression levels of the targeted genes were determined by the 2^−∆∆CT^ method, and *TBP* was used as the control gene. The protein levels of CHK1 (**c**) and p-AKT (**d**) in OSCC patients were assessed by IHC staining. The levels of CHK1 and p-AKT were scored by multiplying the percentage of positive cells (P) by the intensity of staining (I): IHC score = P × I. A box and whisker plot is used to visualize gene expression. The whiskers extend from the 10th percentile to the 90th percentile.

We then examined promising drug targets in the cell cycle and PI3K-AKT pathways and further evaluated drug efficacy in the SAS, OC3, and OEC-M1 cell lines, as well as in CDXs and PDXs. One of the druggable targets, checkpoint kinase 1 (*CHEK1*), is a critical regulator of the DNA damage response (DDR) [22], and was significantly upregulated in the 39 paired OSCC transcriptome datasets and in 305 OSCC patients from TCGA datasets, as shown by RNA-seq (Figure 2a). Upregulation of *CHEK1* in OSCC tumors was further validated by quantitative real-time PCR and immunohistochemical (IHC) staining in 25 paired samples from OSCC patients (Figure 2b,c). The association between clinicopathological characteristics the *CHEK1* was analyzed in another cohort containing 126 paired OSCC samples (Table 2). *CHEK1* overexpression was significantly associated with cervical lymph node metastasis (*p* = 0.032). Cervical lymph nodes are a common site of regional metastasis in OSCC, and cervical lymph node metastasis suggests an increased risk of local or regional recurrence.

The expression of the other druggable targets, *PIK3CA* and *PIK3CD*, which are involved in ECM-receptor interaction and PI3K signaling, was also significantly increased in the 39 paired OSCC transcriptome datasets and in the TCGA datasets, as shown by RNA-seq (Figure 2a). PI3K catalyzes the production of the lipid second messenger phosphatidylinositol-3,4,5-triphosphate (PIP3) and its downstream molecules, including Akt [23]. Hyperactivation of the PI3K-AKT pathway contributes to the proliferation and survival of cancer cells [23]. Upregulation of *PIK3CA* and *PIK3CD* in OSCC tumors was further validated by quantitative real-time PCR in 25 paired samples from OSCC patients (Figure 2b). Phosphorylation of AKT at S473 stimulates full AKT activity, leading to tumor growth [24]^.^ The level of phosphorylated AKT was significantly increased in OSCC tumor tissues compared with adjacent normal tissues, as shown by IHC analysis (Figure 2d). Collectively, these findings demonstrated that *CHEK1*, *PIK3CA*, and *PIK3CD* are significantly upregulated in OSCC. The clinicopathological characteristics related to the expression of *PIK3CA* and *PIK3CD* were analyzed in our 39 OSCC transcriptome datasets (Table S2). However, a significant association was not observed between the expression levels and clinical parameters. Next, we performed a survival analysis to evaluate the roles of these three genes in OSCC prognosis. Kaplan–Meier plots showed the overall survival (OS) for patient subgroups stratified by the levels of *CHEK1*, *PIK3CA*, and *PIK3CD* among the 514 patients in the HNSCC TCGA dataset. Elevated *CHEK1*, *PIK3CA*, and *PIK3CD* levels were not associated with the patients’ overall survival (Figure S4).

### 2.3. Targeting CHK1 and PI3K Decreases Cell Proliferation and Enhances Cell Death in OSCC Cell Lines

**Figure 3:**
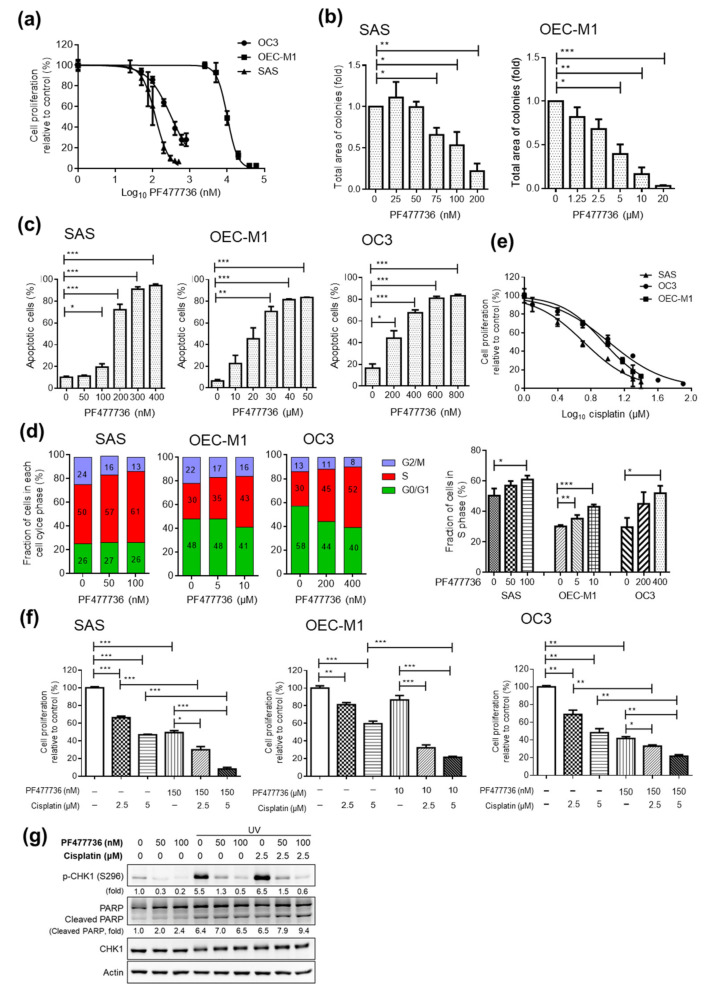
Effect of the CHK1 inhibitor PF477736 alone and in combination with cisplatin in OSCC cell lines. (**a**) Dose–response curve showing the cytotoxic effect of treatment with a CHK1 inhibitor (PF477736) for 48 h in SAS, OC3, and OEC-M1 cells, as assessed by MTT assays. Colony formation assays (**b**) and annexin V staining (**c**) were performed with OSCC cells treated with PF477736 at the indicated concentration. (**d**) The cell cycle distribution of OSCC cells treated with the vehicle control or a CHK1 inhibitor (PF477736) for 24 h at various concentrations was assessed by propidium iodide staining. (**e**) Dose–response curve showing the cytotoxic effect of treatment with cisplatin for 48 h in SAS, OC3, and OEC-M1 cells, as assessed by an MTT assay. (**f**) Cells were treated with PF477736 plus cisplatin for 48 h at the indicated concentrations, and an MTT assay was then performed. The data were replicated in three independent experiments. The results are expressed as the mean ± SEM. values. Statistical analysis of the differences between the two groups was performed with a *t*-test. *p < 0.05; **p < 0.01; ***p < 0.001. (**g**) SAS cells seeded in 6-well plates were exposed to UV radiation (70 mJ/cm^2^) for 2 h. Then, the cells were treated with the CHK1 inhibitor PF477736 and/or cisplatin at the indicated doses for another 2 h. Cell lysates were collected, and Western blot analysis was performed to assess the levels of p-CHK1 (Ser296), PARP, and total CHK1. Actin was used as the loading control.

To evaluate the antitumor effect of the small molecule inhibitors that targeted the two potential proteins, CHK1 and PI3K, we first assessed cell proliferation after CHK1 inhibitor (PF477736, AZD7762, and LY2606368) or PI3K inhibitor (BYL719, GDC0941, and GSK1059615) treatment in OSCC cell lines (SAS, OEC-M1, and OC3). In OSCC cell lines, compared with the vehicle control treatment, the PF477736 treatment decreased cell proliferation, as assessed by MTT assays (Figure 3a). The half-maximal inhibitory concentration (IC_50_) for PF477736 in SAS and OC3 cells ranged from 120 to 320 nM, whereas the IC_50_ concentration in OEC-M1 cells was 10 μM (Figure 3a). The IC_50_ of PF477736 in OEC-M1 cells was significantly higher than that in other OSCC cell lines. However, the protein levels of CHK1 in SAS cells was similar to that in OEC-M1 cells (data not shown), suggesting that the low susceptibility of OEC-M1 cells to the inhibitors may result from the dysregulation of genes involved in multidrug resistance mechanisms.

The colony-forming abilities of SAS and OEC-M1 cells were significantly decreased after PF477736 treatment (Figure 3b). Similar results were observed with two other CHK1 inhibitors (AZD7762 and LY2606368) (Figure S5). Furthermore, cell treated with PF477736 showed significant increases in apoptosis as assessed by annexin V staining (Figure 3c). CHK1 inhibition also significantly induced S phase arrest in the OSCC cell lines (Figure 3d). In addition, to characterize the specificity of these CHK1 inhibitors in SAS cell lines, DNA damage signaling was induced by UV exposure followed by CHK1 inhibitor treatment, and the phosphorylation of CHK1 at Ser296 and the expression level of total CHK1 were analyzed by immunoblotting. These CHK1 inhibitors had specific effects on reducing the phosphorylation of CHK1 (Figure S6 and Figure S7).

Cisplatin is currently the standard treatment for OSCC, and we also tested the effect of cisplatin on OSCC cell lines. Cisplatin significantly decreased the proliferation of OSCC cells, as shown by MTT assays and colony formation assays (Figure 3e and Figure S8). The IC_50_ for cisplatin in the SAS, OC3, and OEC-M1 cell lines ranged from 5 to 10 μM. To evaluate the antitumor effects of combining cisplatin-based chemotherapy with CHK1 inhibitor treatment, we then assessed cell proliferation in OSCC cell lines treated with combinations of cisplatin and PF477736. Interestingly, compared with control and single-agent treatment, combination treatment with cisplatin and PF477736 significantly decreased the proliferation of the OSCC cells (Figure 3f). The Western blot analysis results showed that combination treatment with cisplatin and PF477736 increased PARP cleavage and inhibited CHK1 phosphorylation, suggesting that apoptosis was increased in the SAS cell lines (Figure 3g and Figure S9). Collectively, these results show that both CHK1 inhibition alone or combination treatment with cisplatin and a CHK1 inhibitor effectively decreased cell proliferation, induced cell cycle arrest, and enhanced cell death in the OSCC cell lines.

**Figure 4:**
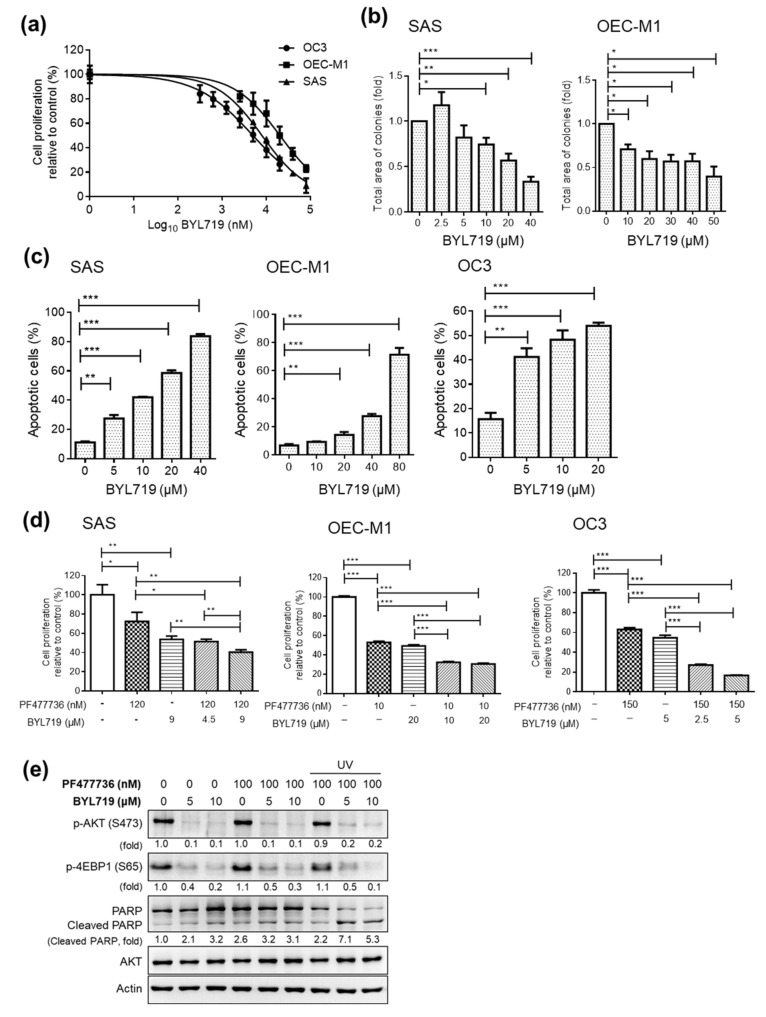
Effect of the PI3K inhibitor BYL719 alone and in combination with the CHK1 inhibitor PF477736 in OSCC cell lines. (**a**) Dose–response curve showing the cytotoxic effect of treatment with the PI3K inhibitor (BYL719) for 48 h in SAS, OC3, and OEC-M1 cells, as assessed by MTT assays. A colony formation assay (**b**) and annexin V staining (**c**) were performed with OSCC cells treated with BYL719 at the indicated concentration. (**d**) Cells were treated with PF477736 plus BYL719 at the indicated concentrations for 48 h, and an MTT assay was then performed. The data were replicated in three independent experiments. The results are expressed as the mean ± SEM values. Statistical analysis of the differences between two groups was performed with a *t*-test. * *p* < 0.05; ** *p* < 0.01; *** *p* < 0.001. (**e**) SAS cells seeded in 6-well plates were exposed to UV radiation (70 mJ/cm^2^) for 2 h. Then, the cells were treated with the CHK1 inhibitor PF477736 and/or the PI3K inhibitor BYL719 at the indicated doses for another 2 h. Cell lysates were collected, and Western blot analysis was performed to assess the levels of p-AKT, p-4EBP1, PARP, and total AKT. Actin was used as the loading control.

The effects of small molecule inhibitors (BYL719, GDC0941, and GSK1059615) targeting the PI3K pathway were also evaluated in this study. The PI3K inhibitor BYL719 significantly decreased cell proliferation, as shown by MTT assays (Figure 4a) and colony formation assays (Figure 4b). Similar results were observed with other PI3K inhibitors (GDC0941 and GSK1059615) (Figure S10). The IC_50_ for BYL719 in the SAS, OC3, and OEC-M1 cell lines ranged from 5 to 20 μM (Figure 4a). Furthermore, the OSCC cell lines treated with BYL719 showed significant increases in apoptosis (Figure 4c). All three PI3K inhibitors effectively inhibited the phosphorylation of AKT at Ser473, as shown by immunoblot analysis (Figure S11 and Figure S12). To evaluate the antitumor effects of the PI3K inhibitor/CHK1 inhibitor combination, we then assessed cell proliferation in OSCC cell lines treated with combinations of BYL719 and PF477736. Interestingly, compared with the control and single-agent treatments, a combination treatment with BYL719 and PF477736 significantly decreased the proliferation of OSCC cells (Figure 4d). Combination treatment with BYL719 and PF477736 also inhibited the phosphorylation of AKT and 4E-BP1 and increased the cleavage of PARP (Figure 4e and Figure S13). These findings raised important questions regarding the antitumor effects of these promising small molecule inhibitors in OSCC preclinical models in vivo.

### 2.4. Antitumor Efficacy of CHK1 Inhibitors and PI3K Inhibitors in SAS Cell Line Xenografts

**Figure 5:**
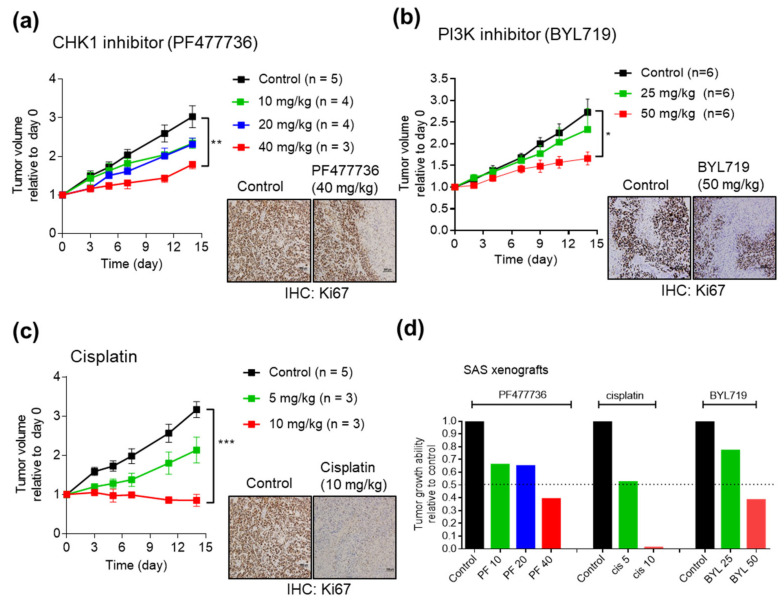
Effects of the CHK1 inhibitor PF477736 and the PI3K inhibitor BYL719 in OSCC cell line-derived xenografts. SAS cells were injected into the subcutaneous tissues of NOD/SCID mice, and when the tumors attained a volume of 300–500 mm^3^, the mice were divided into different experimental groups. Xenografted mice were administered a vehicle control, PF477736 (10, 20, or 40 mg/kg) (**a**); BYL719 (25 or 50 mg/kg) (**b**); or cisplatin (5 or 10 mg/kg) (**c**). The treatment protocols and timelines are described in the Materials and Methods. (**d**) Summary of treatment responses showing the tumor volumes relative to the control. Tumors were measured and tumor volumes were calculated at the indicated times. Ki67 staining was significantly reduced in the treatment group, suggesting a reduction in cancer cell proliferation. The data are expressed as the mean ± SEM values. Statistical analysis of the differences between the two groups was performed with a Student’s *t*-test. * *p* < 0.05; **p < 0.01; *** *p* < 0.001.

To investigate the effect of the CHK1 inhibitors, PI3K inhibitors, and cisplatin in vivo, we established SAS cell line xenografts. SAS cells were injected into the subcutaneous tissues of NOD/SCID mice, and when the tumors attained a volume of 300–500 mm^3^, the mice were divided into different experimental groups. At concentrations of 10 or 20 mg/kg, the CHK1 inhibitor PF477736 partially but not significantly inhibited tumor growth compared with that in the control group (Figure 5a). However, the CHK1 inhibitor PF477736 showed significant antitumor activity at a concentration of 40 mg/kg (Figure 5a). At a dose of 50 mg/kg, the PI3K inhibitor BYL719 significantly inhibited tumor growth compared with that in the control group (Figure 5b). The chemotherapeutic drug, cisplatin inhibited tumor growth compared with that in the control group at a dose of 10 mg/kg (Figure 5c). In addition, the Ki67 staining results showed that PF477736, BYL719, and cisplatin effectively inhibited the growth of SAS CDXs. The tumor volumes in the experimental groups relative to that in the control group were plotted at the experimental endpoint and showed the therapeutic responses in the xenografts (Figure 5d).

### 2.5. Combining CHK1 Targeting with Cisplatin or PI3K Inhibitor Treatment as a Novel Therapeutic Strategy in OSCC PDX Models

**Figure 6:**
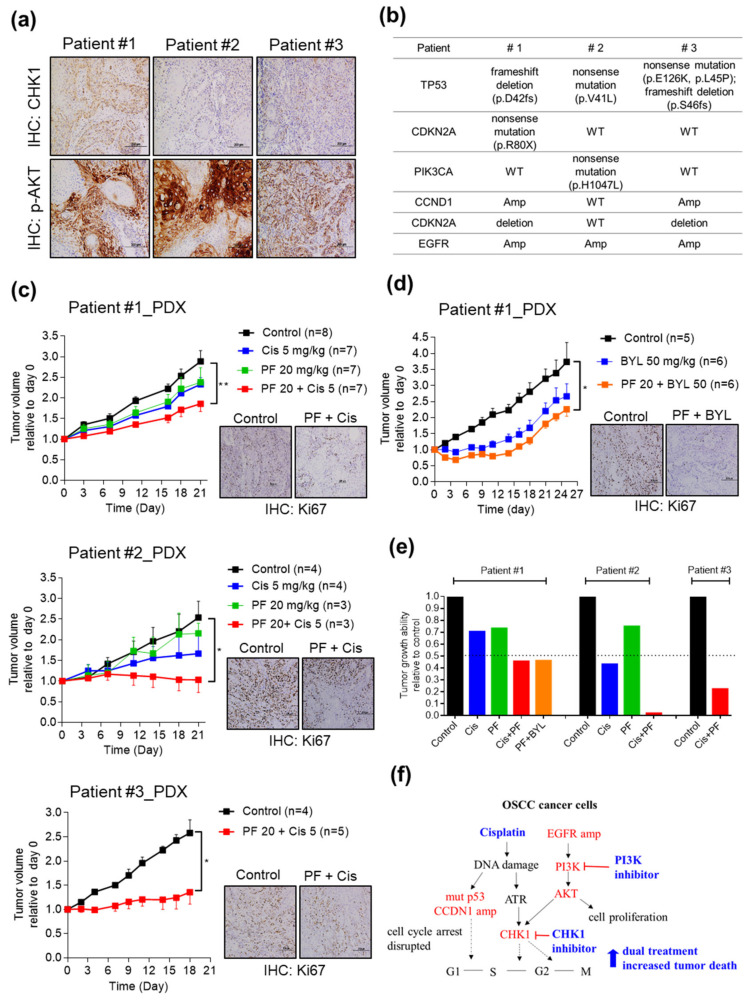
Targeting CHK1 in combination with a PI3K inhibitor or cisplatin treatment synergistically suppresses tumor growth in OSCC PDX models. (**a**) The levels of CHK1 and p-AKT were assessed in OSCC patients. (**b**) Summary of the alterations in *TP53*, *CDKN2A*, *PIK3CA*, *CCND1*, and *EGFR* in OSCC patients. OSCC PDXs (Patient #1 PDX, Patient #2 PDX, and Patient #3 PDX) were established and grouped for different treatments, as follows: control, cisplatin only, CHK1 inhibitor PF477736 only, PF477736 plus cisplatin (**c**), and PF477736 plus BYL719 (**d**). The treatment protocols and timelines are described in the Materials and Methods. (**e**) Summary of treatment responses showing the tumor volumes in the monotherapy and combination therapy groups relative to the control group. Tumor volumes were measured twice weekly, and the mice were monitored for 3 weeks. Ki67 staining was significantly reduced in the combination therapy group, suggesting a reduction in cancer cell proliferation. The data are expressed as the mean ± SEM values. Statistical analysis of the differences between the two groups was performed with a *t*-test. * *p* < 0.05; ** *p* < 0.01. (**f**) The working model, showing the mechanism by which CHK1 inhibition in combination with cisplatin treatment or PI3K inhibition suppresses OSCC growth.

Next, we examined these promising drug targets in OSCC PDXs. First, the expression of CHK1 and phosphorylation of AKT were investigated by IHC analysis in the three OSCC PDX models (Figure 6a). The alterations in *TP53*, *CDKN2A*, *PIK3CA*, *CCND1*, and *EGFR* in the patients are shown in Figure 6b. The PDX models were then divided into a vehicle control group and various treatment groups, including the cisplatin group (5 mg/kg), CHK1 inhibitor (PF477736, 20 mg/kg) group, cisplatin plus PF477736 group, PI3K inhibitor (BYL719, 50 mg/kg) group, and PF477736 plus BYL719 group. The detailed drug treatment protocol is shown in Figure S14a and Figure S15a. Monotherapy with either cisplatin or PF477736 partially inhibited the growth of the PDXs; however, the combination therapy showed synergistic effects on growth inhibition in OSCC PDXs (Figure 6c). Combination therapy with cisplatin and PF477736 showed a statistically significant reduction in the number of Ki67-positive cells, suggesting a reduction in cancer cell proliferation (Figure 6c and Figure S14b).

We then assessed the antitumor efficacy of cotargeting CHK1 and PI3K in OSCC PDXs. Interestingly, BYL719 monotherapy showed a partial but nonsignificant effect on delaying growth, and combination therapy with PF477736 and BYL719 showed significant synergism in slowing tumor growth (Figure 6d). The number of Ki67-positive cells was significantly reduced by combination therapy with PF477736 and BYL719, suggesting a reduction in cancer cell proliferation (Figure 6d and Figure S15b). The tumor volumes in the experimental groups relative to that in the control group were plotted at the experimental endpoint and showed the synergistic antitumor effects of the combination therapy in the PDX models (Figure 6e). Furthermore, the body weight of the mice in the PF477736 and BYL719 combination group was similar to that in the monotherapy group and significant weight loss was not observed in the combined BYL719 plus PF477746 treatment group (Figure S16). Taken together, these data demonstrate that treatment with cisplatin combined with a CHK1 inhibitor or cotargeting CHK1 and PI3K for inhibition are useful novel therapeutic options for OSCC patients with aberrant cell cycle regulation and EGFR-PI3K signaling activation.

## 3. Discussion

In this study, we established OSCC PDX models and characterized the mutational landscapes and dysregulated transcriptome profiles by integrated omics analysis. The genomic signatures and dysregulated genes in the PDX models were comparable to those in the primary tumors. Additionally, these OSCC PDX models recapitulated genomic characteristics similar to those of OSCC, and several genes involved in the cell cycle and PI3K-AKT pathways were consistently upregulated. Cell cycle and PI3K pathway dysregulation have been reported in various tumors, and several clinical trials are currently ongoing to evaluate the clinical efficacy of monotherapies targeting CHK1 or PI3K in head and neck cancer [19,25]. However, the effects of CHK1 and/or PI3K inhibitors in OSCC cell lines and in vivo preclinical animal models are unclear. This observation, together with our studies on the establishment and comprehensive characterization of OSCC PDXs, suggests that PDXs could be used to select suitable personalized treatments and test the efficacy of drugs in individual tumors. As the cell cycle and PI3K pathways were activated in our OSCC cohort, we demonstrated that either monotherapy or dual therapy with CHK1 or PI3K inhibitors would be effective in OSCC cell lines and preclinical models.

CHK1, a serine/threonine-specific protein kinase, regulates the DDR, cell cycle checkpoints, and DNA repair mechanisms [26,27]. CHK1 is regulated by ATM and Rad3-related serine/threonine kinase (ATR) though phosphorylation, forming the ATR-CHK1 cascade, which is involved in the replication checkpoint leading to G2-phase arrest. Many reports indicate that ATR-CHK1 activation may promote tumor growth and that *CHEK1* expression is upregulated in triple-negative breast cancer, neuroblastoma, T-cell leukemia, nasopharyngeal cancer, ovarian cancer, and glioblastoma [28,29,30,31,32,33]. In HNSCC, CHK1 phosphorylation is significantly elevated in tumors [33]. Parikh et al. showed by quantitative PCR that both *ATR* and *CHEK1* are overexpressed in 7 OSCC cell lines [34]. We first identified that *CHEK1* was significantly overexpressed in clinical specimens and that elevated *CHEK1* expression was positively correlated with cervical metastasis in 126 OSCC patients. The efficacy of CHK1 inhibitor monotherapy or combination therapy with CHK1 inhibitors and cisplatin or docetaxel has been explored in clinical trials with enrolled patients with advanced solid tumors, breast cancer, and ovarian cancer [25,35]. Additionally, genetic studies have reported that *TP53* is the most frequently mutated gene in oral cancer and that *TP53* mutation is a prognostic biomarker for poor outcomes in head and neck cancer [36]. Additionally, targeting *TP53* mutant cells with CHK1 inhibitors sensitized these cells to genotoxic agents in a cell line platform [37]. In the present study, PDXs from all three patients with *TP53* mutations exhibited an effective therapeutic response to synergistic treatment with the CHK1 inhibitor PF477736 and cisplatin. Therapeutically, the efficacy of combination therapy with a CHK1 inhibitor and cisplatin in our preclinical OSCC PDX models with cell cycle dysregulation and *CHEK1* overexpression suggested the promise of this therapeutic approach in OSCC patients. Recently, an association between genetic deletion of cyclin-dependent kinase inhibitor 2A (*CDKN2A*/*p16*) and sensitivity to Chk1 inhibition was reported in HNSCC cell lines [38]. The p.R80X *CDKN2A* mutation or *CDKN2A* deletion was detected in OSCC Patients #1 and #3 but not in Patient #2. The therapeutic response to combination therapy with cisplatin and the CHK1 inhibitor was significantly stronger than that to monotherapy in Patient #2, suggesting that CHK1 targeting combined with cisplatin treatment may also be beneficial in patients with wild-type *CDKN2A*. Moreover, other molecules involved in cell cycle regulation may have been altered, and more models with/without mutation of only *CDKN2A* must be tested.

The PI3K-AKT signaling pathway is another pathway dysregulated in head and neck cancer, leading to growth factor-independent cell proliferation in tumors [3,39]. In this report, we found that *PIK3CA* and *PIK3CD* were overexpressed in OSCC clinical specimens. Additionally, the PI3K inhibitors (BYL719, GDC0941, and GSK1059615) can inhibit cell proliferation and induce cell death in OSCC cell lines. Alpelisib (BYL719) is a PI3K alpha-specific inhibitor with antitumor activity in vitro and in vivo [19,40,41]. PI3K-targeted monotherapies are currently in clinical development for the treatment of solid tumors [19]. Interestingly, our results demonstrated that the combination of BYL719 and PF477736 showed synergistic effects both in vitro and in vivo. In the present study, BYL719 effectively inhibited the downstream AKT signaling and 4E-BP1 phosphorylation in OSCC cells. Additionally, cotreatment with BYL719 and PF477736 more effectively inhibited downstream signaling activation and increased PARP cleavage than treatment with either agent as monotherapy.

Kinase inhibitors may produce off-target effects by inducing changes in molecules other than the specifically targeted molecule. For instance, PF477736, one of the CHK1 inhibitors used here, can target VEGFR2, Aurora A, and CHK2 [42]; however, PF477736 shows an approximately 30- to 100-fold higher selectivity for CHK1 than other targets [42]. LY2606368 and AZD7762 are two additional specific CHK1 inhibitors and show antitumor effects in OSCC. GSK1059615 is a dual inhibitor of PI3K and mTOR [43] and shows inhibitory activity in OSCC along with two other PI3K inhibitors (BYL719 and GDC0941). Collectively, these results suggest that inhibition of OSCC growth may primarily be associated with the targeting of CHK1 and PI3K.

Clinically, if OSCC patients undergo genetic testing to identify increased expression of *CHEK1*, *PIK3CA*, or *PIK3CD*, combination therapy with a CHK1 inhibitor and a PI3K inhibitor could be suggested for consideration.

## 4. Materials and Methods

### 4.1. Patient Characteristics and Clinical Specimens

OSCC patients were enrolled at Chang Gung Memorial Hospital, Taiwan. This study was approved by the Institutional Review Board at Chang Gung Memorial Hospital (Protocol No: 102-5685A3), Taiwan. Prior to sample collection, written informed consent was obtained from all participants. Primary tumors from three late-stage OSCC patients were successfully transplanted and expanded into immunodeficient mice (Table 1). Tumor and adjacent normal tissues from 27 OSCC patients in the first cohort were collected for IHC staining and quantitative reverse transcription polymerase chain reaction (qRT-PCR) analysis (Table S1). Tumor specimens were obtained from another 126 OSCC patients in the second cohort for *CHEK1* qRT-PCR analysis and univariable analysis (Table 2).

### 4.2. Cell Culture

SAS cells were maintained in Dulbecco’s modified Eagle’s medium (DMEM; Gibco, USA) containing 100 U/mL penicillin and 100 μg/mL streptomycin and supplemented with 10% fetal bovine serum (FBS; Gibco, USA). OEC-M1 cells were maintained in Roswell Park Memorial Institute (RPMI) 1640 medium (Thermo Fisher Scientific, USA), and OC3 cells were maintained in DMEM supplemented with keratinocyte serum-free medium (KSFM; Gibco, USA) and containing antibiotics and 10% FBS. All cell lines were cultured at 37°C in a humidified atmosphere containing 5% CO_2_.

### 4.3. Drugs, Chemicals, and Antibodies

The CHK1 inhibitors PF477736 (Sigma-Aldrich, USA), AZD7762 (Selleck Chemicals, CA, USA), and LY2606368 (MedChem Express, USA); as well as the PI3K inhibitors GDC0941 (Cayman Chemical, USA) and GSK1059615 (Cayman Chemical, USA) were used. The PI3K inhibitor BYL719 was kindly provided by Novartis Pharma AG (Basal, Switzerland). Cisplatin was purchased from Sigma-Aldrich. Antibodies against the following molecules were used for immunoblotting: CHK1, p-CHK1-Ser296, AKT, p-AKT-Ser473 (Cell Signaling Technology), and GAPDH (Bioworld, USA).

### 4.4. RNA-Seq and Transcriptome Analysis

Total RNA from the paired tumor tissues and xenografts was extracted using TRIzol Reagent (Gibco BRL), and the RNA quality and quantity were confirmed using an Agilent 2100 Bioanalyzer (Agilent Technologies, Santa Clara, CA). For RNA-seq, libraries were prepared using the TruSeq Stranded mRNA Sample Preparation Guide (Part # 15031047 Rev. E; Illumina) according to the manufacturer’s instructions. Sequencing was conducted in an Illumina NextSeq 500 instrument. Transcriptome and pathway analyses were performed as previously described [44]. Cancer hallmark enrichment analysis was performed, and cancer hallmark definitions were downloaded from the GSEA/MSigDB 6.2 (http://www.broadinstitute.org/gsea/msigdb) database; the hallmarks were screened to include only those highly linked to cancer according to Drake et al [45]. Enrichment analysis was performed by calculating the probability of overlap between the test set (defined as the set of genes in a network model) and the hallmark sets using the DoGsea function of the Bioconductor package clusterProfiler (3.12.0) [46]. Hallmark “wheels” are colored proportionally to the negative log *p* values (or normalized enrichment scores) returned by the DoGsea function.

### 4.5. Whole-Exome Sequencing

Genomic DNA was extracted from tumor and normal tissues using the QIAamp DNA Mini Kit (Qiagen) in accordance with the manufacturer’s protocols. The quality and DNA concentrations of the samples were assessed with a Qubit fluorometer (Thermo Fisher Scientific) and 0.8% agarose gel electrophoresis. For whole-exome sequencing and bioinformatic analysis, high-quality genomic DNA was captured using a SureSelect Human All Exon V6 + COSMIC Kit according to the manufacturer’s protocol (Agilent Technologies) and analyzed as previously reported [6,44].

### 4.6. Quantitative Real-Time PCR (qRT-PCR)

For quantitative PCR, first-strand complementary DNA was synthesized from 1 μg of total RNA using random hexamer primers (GeneDirex, Germany) and SuperScript III RT (Invitrogen, USA). The sequences of the primers were as follows: CHEK1-F (5’-TCGGTATAATAATCGTGAGCG-3’), CHEK1-R (5’-ACAGGACCAAACATCAACTG-3’), PIK3CA-F (5’-ACGATGGACAACTGTTTCA-3’), PIK3CA-R (5’-GTCTTTGTGCATTCTTGGG-3’), PIK3CD-F (5’-GACATCCAGTATCTCAAGGAC-3’), PIK3CD-R (5’-AGCCAGTTCACTTTGGTT-3’), TBP-F849 (5’-TGCTCACCCCACCAACAATTTAG-3’), and TBP-R969 (5’-CTGGGTTTGATCATTCTGTAGATTAA-3’).

### 4.7. Cell Cycle Analysis and Cell Viability Assay

For cell cycle analysis, cells were treated as indicated for 24 h. Cells were fixed with 70% ethanol, stained with 50 µg/mL propidium iodide (Sigma-Aldrich, USA) and analyzed by flow cytometry. For the cell viability assay, cells were treated for 48 h. MTT (5 mg/mL) was then added to each well for the MTT cell viability assay. After incubation for 1 h, the supernatant was discarded, DMSO (100 μL) was added, and the absorbance was measured at 540 nm in a spectrophotometer (SpectraMax M2; Molecular Devices, USA). For the apoptosis assay, cells were treated at the indicated concentrations for 48 h and were then stained with a FITC-conjugated Annexin V antibody (Invitrogen, USA) and propidium iodide (Sigma-Aldrich, USA). Cells were evaluated by flow cytometry (Attune NxT; Invitrogen, USA), and the data were analyzed with FlowJo software (Tree Star, Inc.).

### 4.8. Immunoblotting

Total protein was extracted with RIPA lysis buffer (1% NP-40, 20 mM Tris-HCl, 150 mM NaCl, 1 mM Na_3_VO_4_, 5 mM EDTA, 10% glycerol, PMSF, and 0.2% protease inhibitor). Equal amounts of protein were separated by SDS-PAGE and transferred to a PVDF membrane, which was incubated with the appropriate primary antibodies. Immunoreactions were detected with HP-conjugated secondary antibodies and ECL substrate (PerkinElmer, USA).

### 4.9. IHC Staining

Tissue sections were subjected to antigen retrieval using Bond Epitope Retrieval Solution 2 in a Bond-Max automated immunostainer (Leica Biosystems) and stained with antibodies against CHK1 (Abcam), phosphorylated AKT (Abcam), and Ki67 (Cell Signaling). The remaining steps were performed in accordance with standard procedures.

### 4.10. PDX Establishment and Dug Sensitivity Tests in Vivo

NOD/SCID mice (obtained from the National Library Animal Center, Taiwan) and NOD.Cg-Prkdc^scid^ Il2rg^tm1Wjl^/SzJ (NSG) mice (obtained from The Jackson Laboratory) were housed in a specific-pathogen-free animal room. All animal experiments were approved by the Institutional Animal Care and Use Committee of Chang Gung University (Protocol Nos: CGU106-114 and CGU107-074). For PDX establishment, patient tumor explants were obtained from surgical specimens and engrafted in NSG mice within 2 h. In brief, fresh tumor tissues were rinsed in PBS containing an antibiotic-antimycotic solution (Gibco, USA) and cut into small pieces (~1 mm^3^). Each NSG mouse was subcutaneously inoculated with a tumor fragment weighing 50–100 mg in the left flank to establish the first-generation (P1) PDX. When the tumors attained an average volume of 1000 mm^3^, they were excised and were then transplanted and expanded to establish the next generation (P2). When the tumors attained an average volume of 300–500 mm^3^, the mice were assigned randomly to the control and various experimental groups. For drug tests in the PDX models, 20 mg/kg PF477736 was administered by intraperitoneal injection (4 days in the first week). BYL719 (50 mg/kg) was administered by oral gavage (5 days/week for two weeks). Cisplatin (5 mg/kg) was administered by intraperitoneal injection (2 days in the first week). To establish the SAS cell line-derived xenografts, 100 µL of a SAS cell suspension (5 × 10^6^ cells per ml) was injected subcutaneously into NOD/SCID mice, and tumors were established after 3–4 weeks. When the xenografts attained a volume of 300–500 mm^3^, the mice were randomly divided into the experimental and control groups. PF477736 (10, 20, or 40 mg/kg) was administered by intraperitoneal injection (4 days in the first week). BYL719 (25 or 50 mg/kg) was administered by oral gavage (5 days/week for two weeks). Cisplatin (5 or 10 mg/kg) was administered by intraperitoneal injection (2 days in the first week). Tumors were measured with calipers, and the volume was calculated as follows: volume = 1/2 × length × width^2^.

### 4.11. Statistical Analysis

The Wilcoxon test was used to analyze the qRT-PCR results for OSCC and normal counterpart tissues. Between-group comparisons were performed with Student’s *t*-tests or the Mann–Whitney *U* tests. All statistical tests were performed with SPSS software version 12.0 (SPSS, Inc., Chicago, IL, USA) or Prism. A *p* value of < 0.05 was considered to indicate a statistically significant difference.

## 5. Conclusions

In this study, OSCC PDX preclinical models were established, and their therapeutic implications were explored. Notably, the expression levels of *CHEK1*, *PIK3CA*, and *PIK3CD* were significantly increased in both the primary tumor tissues and xenografts. The antitumor efficacy of CHK1 inhibitors and PI3K inhibitors was validated in vitro and in vivo. Targeting either CHK1 or PI3K effectively induced cell cycle arrest and apoptosis in OSCC cells. Combination therapy with cisplatin-based chemotherapy and a CHK1 inhibitor exhibited a synergistic antitumor effect. Furthermore, combination therapy with CHK1 and PI3K inhibitors resulted in greater therapeutic efficacy than treatment with either agent as monotherapy via suppression of CHK1, AKT, and 4E-BP1 phosphorylation. Overall, our results provide proof of principle for CHK1 inhibition in combination with either PI3K inhibition or cisplatin treatment as a novel therapeutic strategy for OSCC (Figure 6f).

## Figures and Tables

**Table 1:** Characteristics of the oral cavity squamous cell carcinoma (OSCC) patients enrolled for the patient-derived xenograft (PDX) model establishment.

Characteristics	Patients		
	#1	#2	#3
Age (years)	47	60	66
Sex	M	M	M
Tumor classification	4A	2	4A
Node classification	2B	2B	0
Overall TNM stage	IV	IV	IV
Differentiation ^a^	Moderate	Moderate	Moderate
Site	Tongue	Buccal mucosa	Other
Alcohol drinking	Y	Y	Y
Betel quid chewing	Y	Y	Y
Cigarette smoking	N	Y	Y

^a^ Well: well-differentiated squamous cell carcinoma; Moderate: moderately differentiated squamous cell carcinoma.

**Table 2:** Associations of the clinicopathological characteristics with CHEK1 expression levels in the 126 OSCC patients in this study.

Patient Characteristics	Number of Patients	No. of Patients (%)		*p* Value
		*CHEK1* Low Expression	*CHEK1* High Expression	
**Sex**				
Male	114	57 (50.0)	57 (50.0)	1.000
Female	12	6 (50.0)	6 (50.0)	
**Age ^a^** (year)		51.8 ± 11.3	52.8 ± 1.2	0.336
**Tumor classification**				
T1–T2	63	33 (52.4)	30 (47.6)	0.593
T3–T4	63	30 (47.6)	33 (52.4)	
**Node classification**				
N = 0	64	38 (59.4)	26 (40.6)	0.032 ^b^
N > 0	62	25 (40.3)	37 (59.7)	
**Overall TNM stage**				
I–II	38	23 (60.5)	15 (39.5)	0.120
III–IV	88	40 (45.5)	48 (54.5)	
**Extranodal extension**				
No	94	49 (52.1)	45 (47.9)	0.413
Yes	32	14 (43.8)	18 (56.2)	
**Perineural invasion**				
No	68	36 (52.9)	32 (47.1)	0.475
Yes	58	27 (46.6)	31 (53.4)	
**Differentiation ^c^**				
Well + Mod	110	57 (51.8)	53 (48.2)	0.285
Poor	16	6 (37.5)	10 (62.5)	
**Tumor depth**				
mm		14.3 ± 9.5	13.5 ± 10.3	0.475

^a^ Mean ± SD. ^b^ Statistically significant. ^c^ Well: well-differentiated squamous cell carcinoma; Mod: moderately differentiated squamous cell carcinoma; Poor: poorly differentiated squamous cell carcinoma.

## Supplementary Materials

The following are available online at https://www.mdpi.com/2072-6694/12/7/1726/s1, Figure S1: Transcriptome alterations in OSCC tumors; Figure S2: Enriched cancer hallmarks in three OSCC patients; Figure S3: Pathway enrichment analysis of 39 paired OSCC transcriptome datasets; Figure S4. Kaplan-Meier survival curves for overall survival according to the levels of CHEK1, PIK3CA, and PIK3CD in HNSCCs; Figure S5: Screening of CHK1 inhibitors in OSCC cell lines; Figure S6: CHK1 inhibitors effectively inhibit the UV-induced phosphorylation of CHK1; Figure S7: Uncropped scans of Western blots in Figure S6; Figure S8: Effects of cisplatin in OSCC cell lines, Figure S9. Uncropped scans of Western blots in Figure 3g; Figure S10: Screening of PI3K inhibitors in OSCC cell lines; Figure S11: PI3K inhibitors effectively inhibit the phosphorylation of AKT; Figure S12: Uncropped scans of Western blots in Figure S11; Figure S13: Uncropped scans of Western blots in Figure 4e; Figure S14: PF477736 and cisplatin treatment in OSCC PDX models; Figure S15: BYL719 and PF477736 treatment in OSCC PDX models; Figure S16: Body weight of OSCC PDX models upon treatment. Table S1: Clinical characteristics of 27 OSCC patients in the first cohort; Table S2: Clinical characteristics related to PIK3CA and PIK3CD expression in the OSCC transcriptome datasets.
