# Supplementary material for: Cotargeting CHK1 and PI3K Synergistically Suppresses Tumor Growth of Oral Cavity Squamous Cell Carcinoma in Patient-Derived Xenografts
Source: Cancers (Basel). 2020 Jun 29;12(7):1726. doi: 10.3390/cancers12071726 (PMC7408003; doi:10.3390/cancers12071726)
Supplement: Supplementary file 1 [file cancers-12-01726-s001.pdf]

cancers

MDPI

Article

Cotargeting CHK1 and PI3K Synergistically Suppresses Tumor Growth of Oral Cavity Squamous Cell Carcinoma in Patient-Derived Xenografts

Chia-Yu Yang, Chiao-Rou Liu, Ian Yi-Feng Chang, Chun-Nan OuYang, Chia-Hsun Hsieh, Yen-Lin Huang, Chun-I Wang, Fei-Wen Jan, Wan-Ling Wang, Ting-Lin Tsai, Hsuan Liu, Ching-Ping Tseng, Yu-Sun Chang, Chih-Ching Wu, Kai-Ping Chang

Supplementary Materials:

Table S1. Clinical characteristics of 27 OSCC patients in the first cohort.

Characteristics	Stage of OSCC	
	Stage I-II	Stage III-IV
Number of patients	3	24
**Age (years)**		
Range	44 ~ 84	35 ~ 66
Mean ± SD	63.4 ± 20.3	50.6 ± 10.3
**Sex**		
Male	2 (66.7%)	21 (87.5%)
Female	1 (33.3%)	3 (12.5%)
**Tumor classification**		
T1-T2	3 (100.0%)	8 (33.3%)
T3-T4	0 (0.0%)	16 (66.7%)
**Node classification**		
N(-)	3 (100.0%)	7 (29.2%)
N(+)	0 (0.0%)	17 (70.8%)
**Overall TNM stage**		
I	0 (0.0%)	-
II	3 (100.0%)	-
III	-	5 (20.8%)
IV	-	19 (79.2%)
**Alcohol drinking**		
No	2 (66.7%)	5 (20.8%)
Yes	1 (33.3%)	19 (79.2%)
**Betel quid chewing**		
No	1 (33.3%)	5 (20.8%)
Yes	2 (66.7%)	19 (79.2%)
**Cigarette smoking**		
No	1 (33.3%)	4 (16.7%)
Yes	2 (66.7%)	20 (83.3%)

Table 2. Clinical characteristics related to *PIK3CA* and *PIK3CD* expression in the OSCC transcriptome datasets.

Patient categories	Case No. (%)	Gene Expression Level (Normalized Read Counts)	
		PIK3CA	*PIK3CD*
**Tumor classification**			
T1-T2	13 (33.3%)	33	24
T3-T4	26 (67.7%)	34	30
p value		0.7471	0.2829
**Node classification**			
N = 0	22 (56.4%)	33	25
N > 0	17 (43.6%)	35	32
p value		0.6736	0.1950

**Overall TNM stage**			
I-II	9 (23.1%)	30	24
III-IV	30 (76.9%)	35	29
p value		0.2504	0.4203

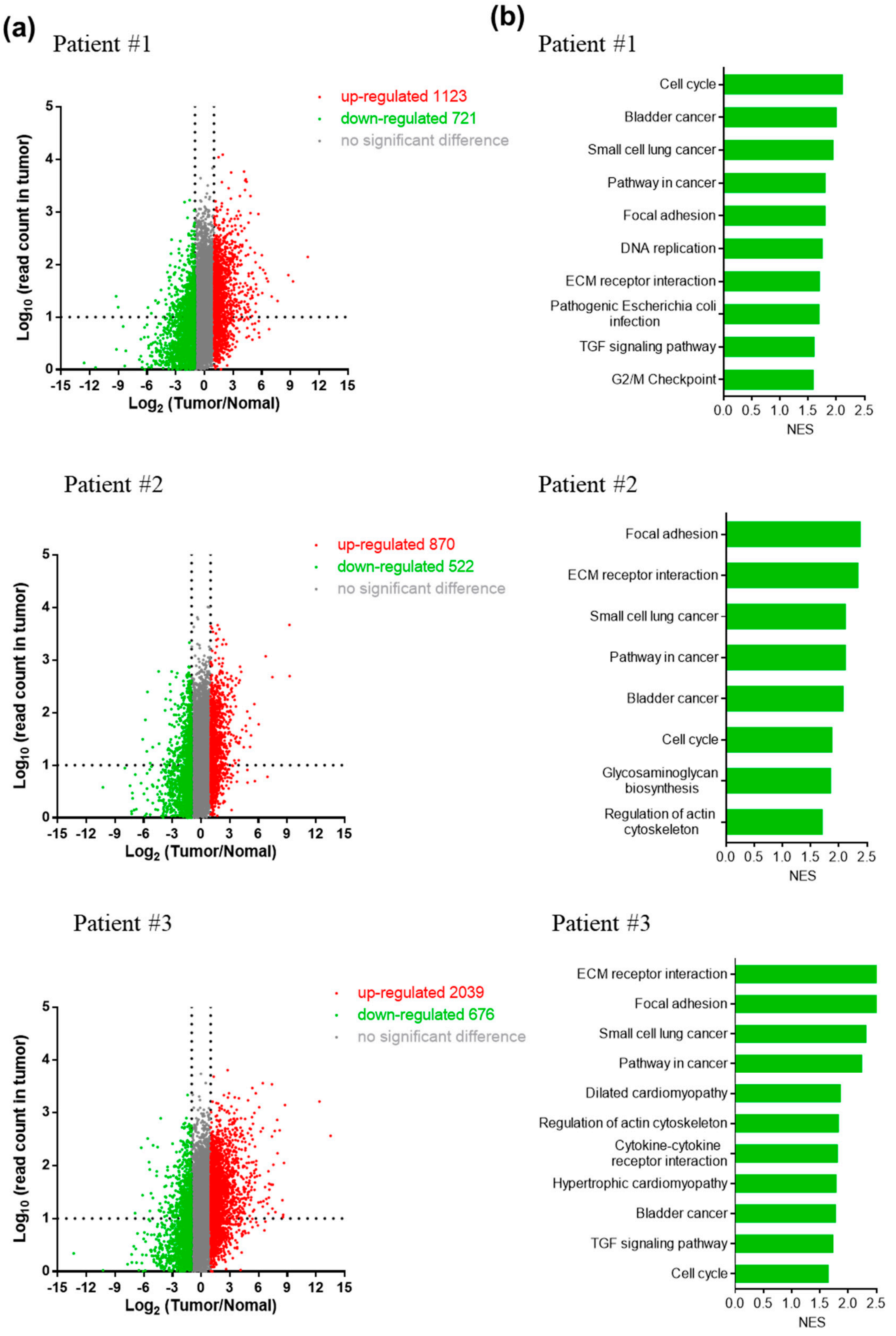

Figure S1. Transcriptome alterations in OSCC tumors. RNA-seq analysis to identify DEGs in OSCC. RNA deep sequencing was performed on 3 pairs of OSCC tumor samples and adjacent normal tissues.

Upon read alignment, gene expression levels were determined based on the normalized read count values. (**a**) Volcano plots generated by differential gene expression analysis in OSCC tumor and adjacent normal tissues. The x-axis shows the log2(fold change) values, and the y-axis shows the log10(normalized read count) values for the differentially expressed genes. (**b**) The dysregulated genes in individual OSCC clinical samples (paired tumor and adjacent normal tissues) were subjected to GSEA. The significantly upregulated pathways in tumors are shown.

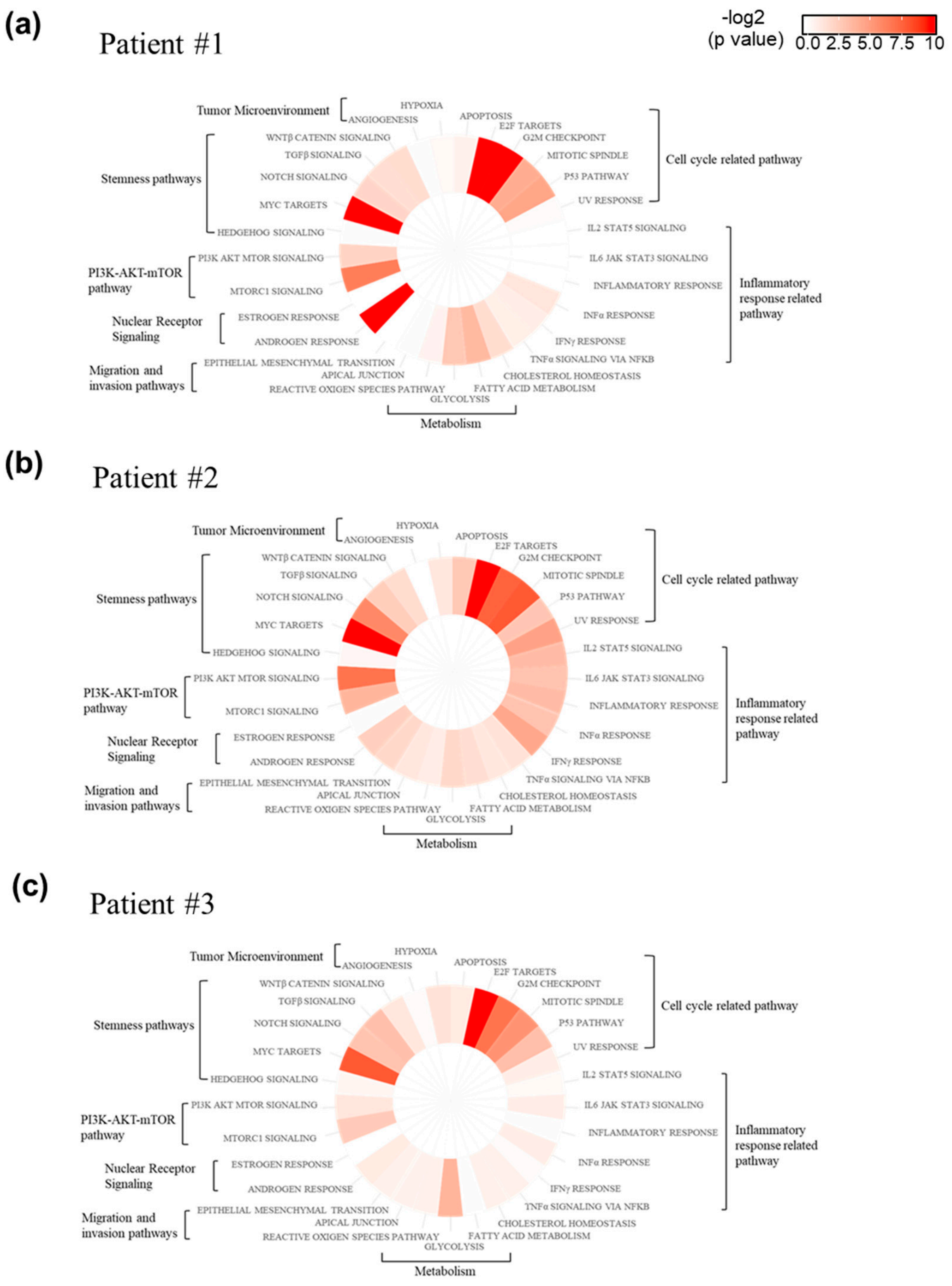

Figure S2. Enriched cancer hallmarks in three OSCC patients. The transcriptome profiles of OSCC patient tumors were determined by RNA-seq. The DEGs in patient #1 (**a**), patient #2 (**b**), and patient #3 (**c**) were subjected to cancer hallmark enrichment analysis. The hallmark wheels show enrichment of several different pathways, including cell cycle-related pathways and the PI3K-AKT-mTOR pathway, in individual patients.

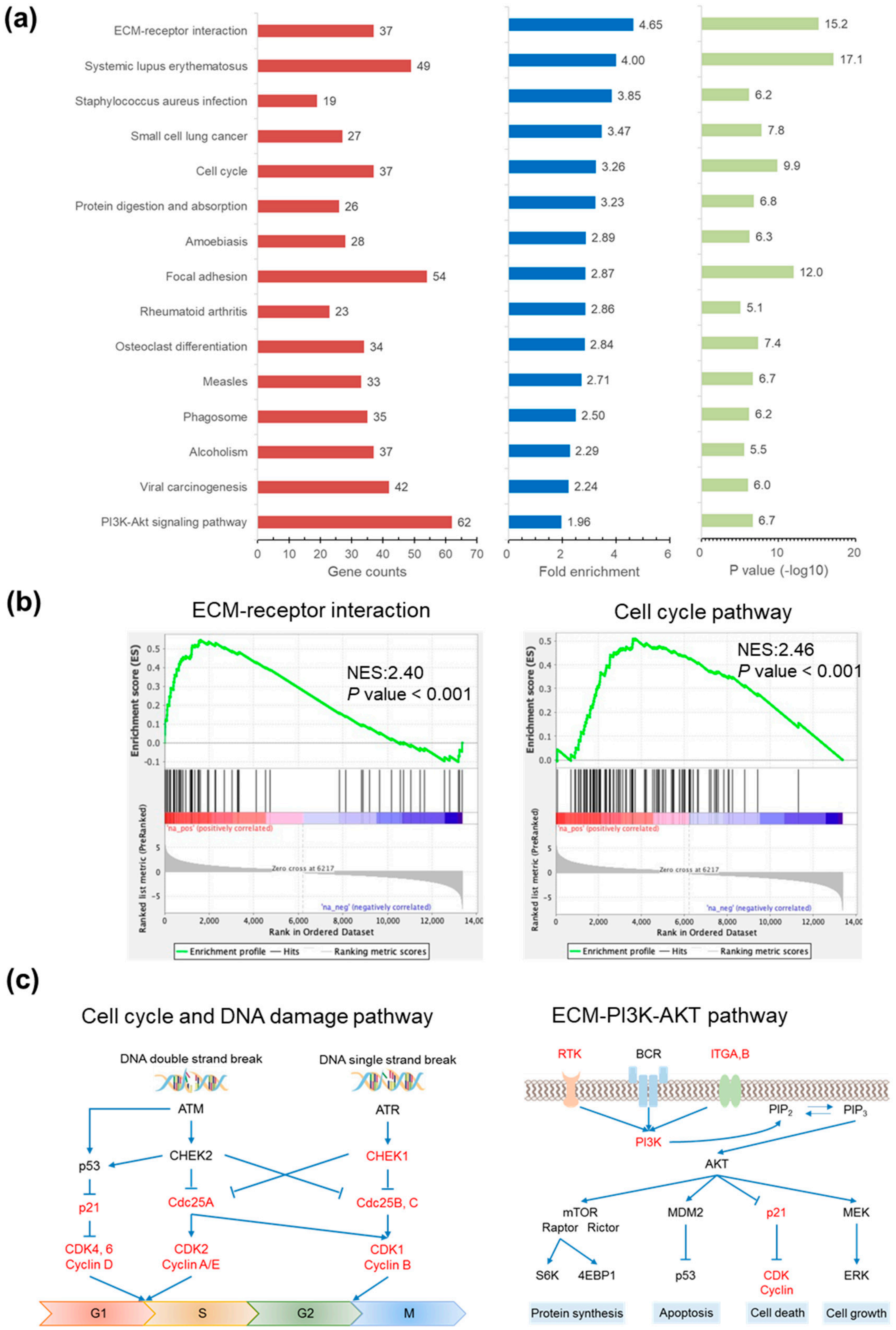

Figure S3. Pathway enrichment analysis of 39 paired OSCC transcriptome datasets. The dysregulated genes in 39 paired OSCC transcriptome datasets were analyzed with DAVID (**a**) and GSEA (**b**). The significantly upregulated pathways, gene counts, *p* values, and NESs in tumors are shown. (**c**) The cell cycle and DNA damage pathway (left panel) and the PI3K-AKT pathway (right panel) were selected, and the significantly upregulated genes in tumors are shown in red.

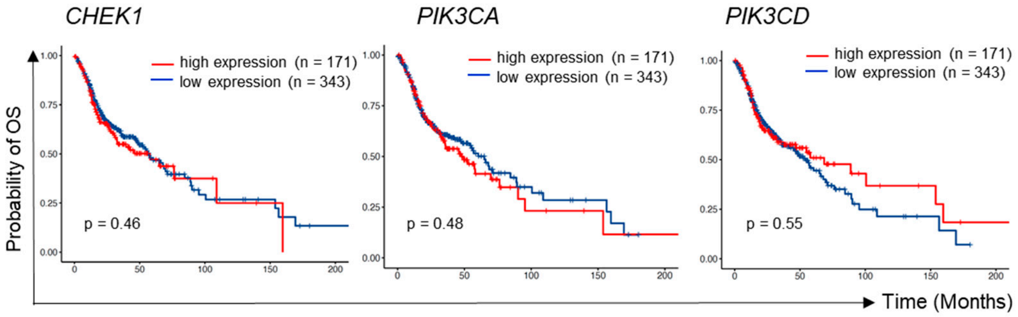

Figure S4. Kaplan-Meier survival curves for overall survival according to the levels of *CHEK1*, *PIK3CA*, and *PIK3CD* in HNSCCs. Kaplan-Meier plot showing the overall survival for patient subgroups stratified by high versus low gene expression among the 514 patients in the HNSCC-TCGA data set. The *p* values were calculated using log-rank tests.

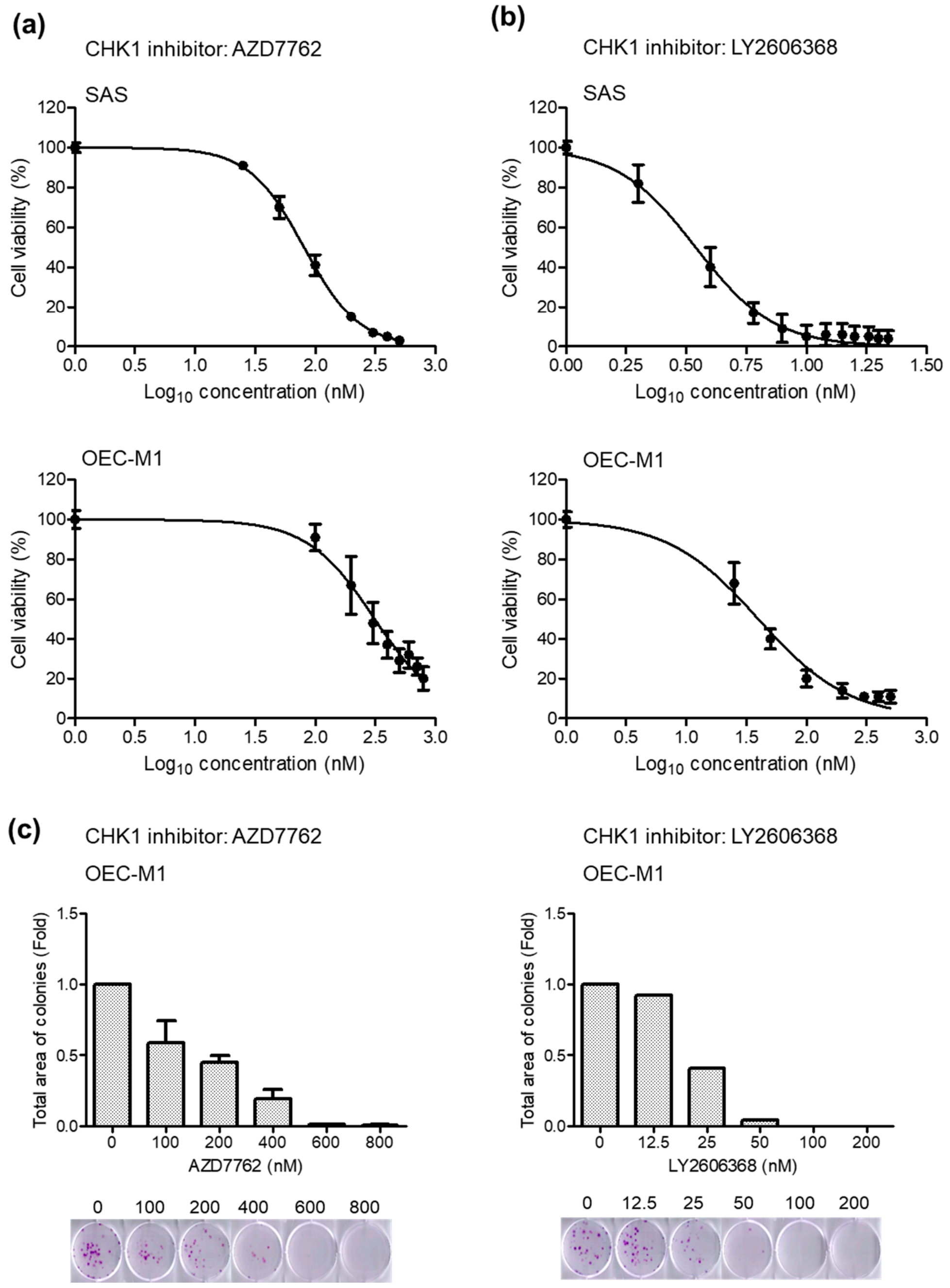

Figure S5. Screening of CHK1 inhibitors in OSCC cell lines**.** OSCC cells seeded in 96-well plates were treated in triplicate with CHK1 inhibitors at various concentrations for 2 days. After inhibitor treatment, cells were subjected to an MTT assay. The dose-response curve shows the cytotoxic effect of the CHK1 inhibitors AZD7762 (**a**) and LY2606368 (**b**) in SAS and OEC-M1 cells. (**c**) Colony formation assays of OEC-M1 cells treated with AZD7762 or LY2606368 at the indicated concentrations.

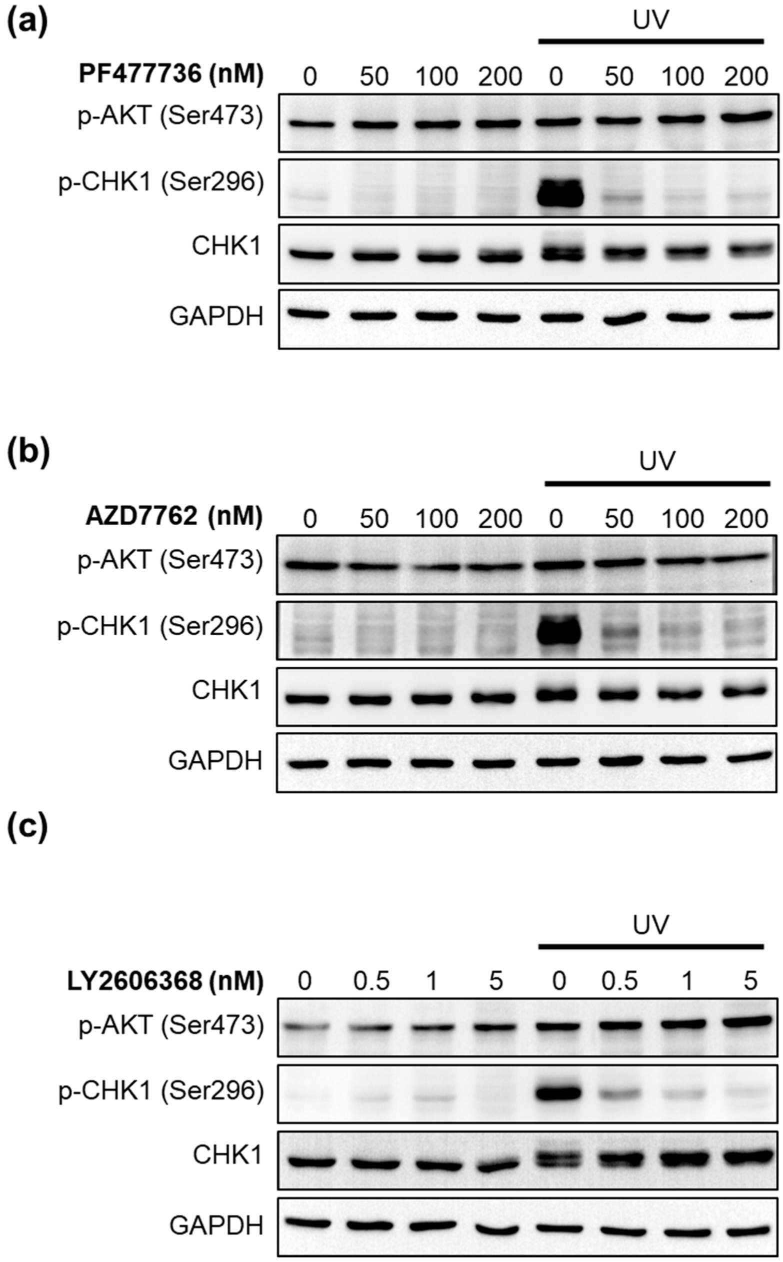

Figure S6. CHK1 inhibitors effectively inhibit UV-induced phosphorylation of CHK1. SAS cells seeded in 6-well plates were exposed to UV radiation (70 mJ/cm^2) for 2 h. Then, the cells were treated with DMSO only or with the CHK1 inhibitors PF477736 (**a**), AZD7762 (**b**), and LY2606368 (**c**) at the indicated doses for another 2 h. Cell lysates were collected, and Western blot analysis was performed to assess the levels of pCHK1 (Ser296), p-AKT (Ser4730) and total CHK1. GAPDH was used as the loading control.

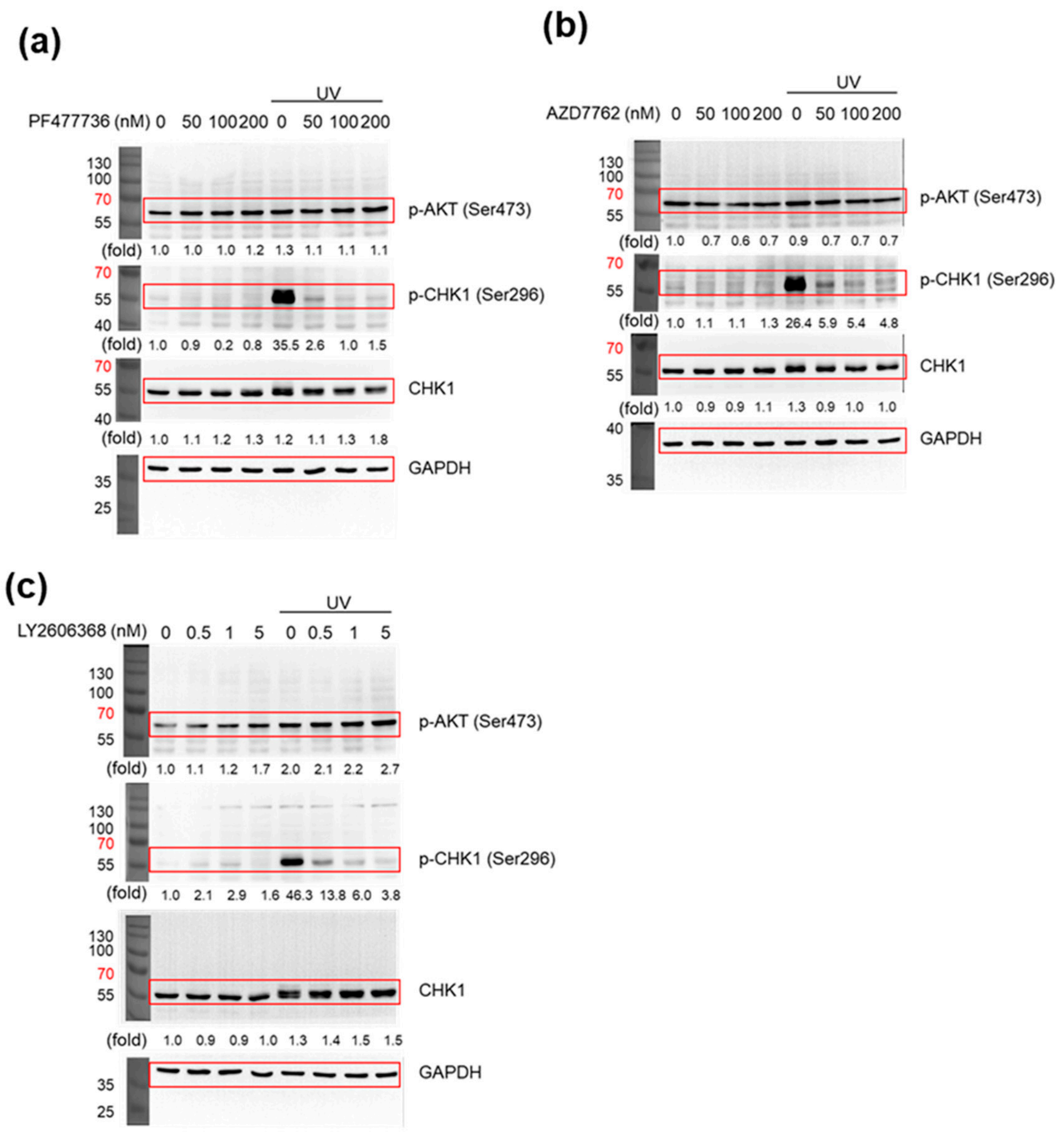

Figure S7. Uncropped scans of Western blots in Figure S6. In each lane, the band intensity ratios of p-CHK1, p-AKT, and CHK1 to GAPDH was calculated. The ratios in the untreated sample were set to 1.

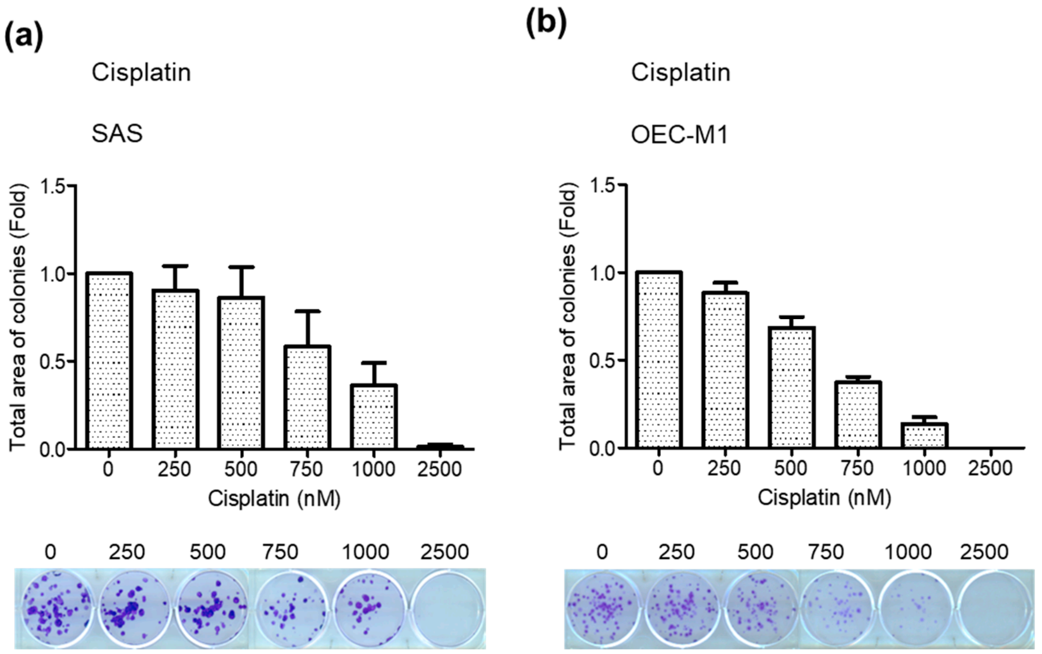

Figure S8. Effects of cisplatin in OSCC cell lines. Colony formation assays of SAS **(a)** and OEC-M1 **(b)** cells treated with cisplatin at the indicated concentrations.

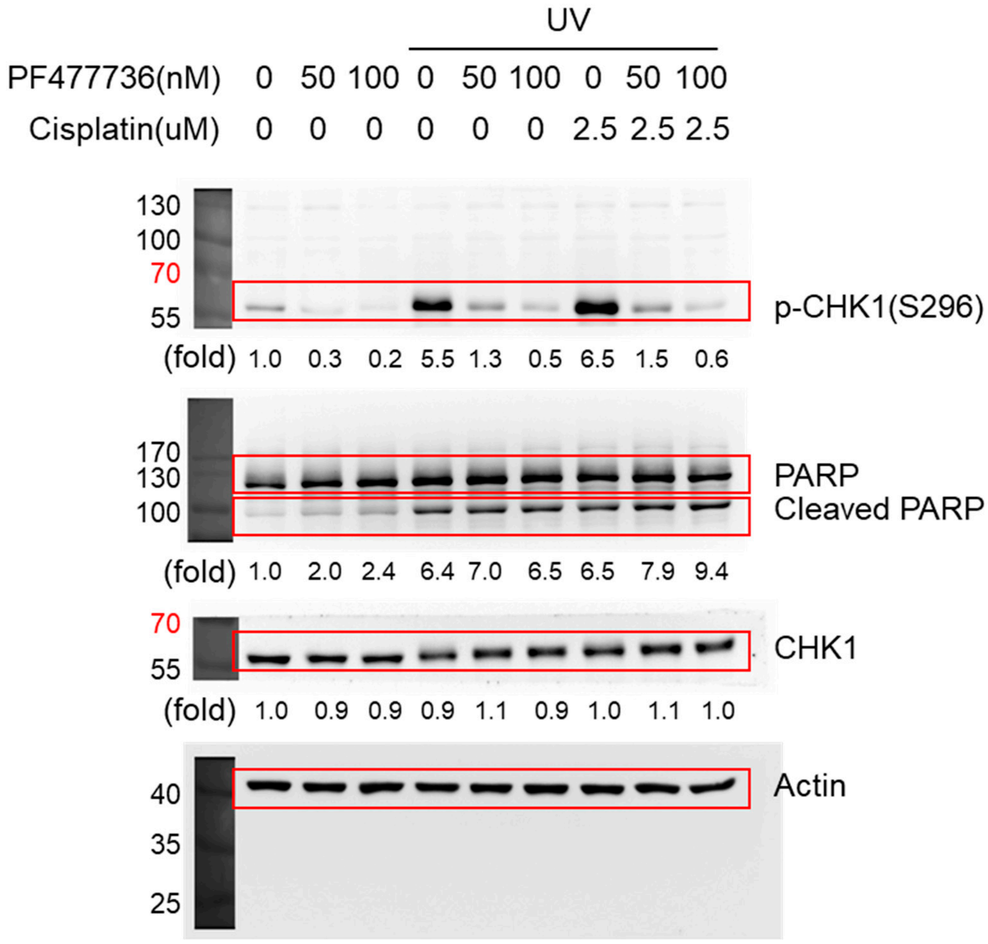

Figure S9. Uncropped scans of Western blots in Figure 3g. In each lane, the band intensity ratios of p-CHK1, cleaved PARP, and CHK1 to Actin were calculated. The ratios in the untreated sample were set to 1.

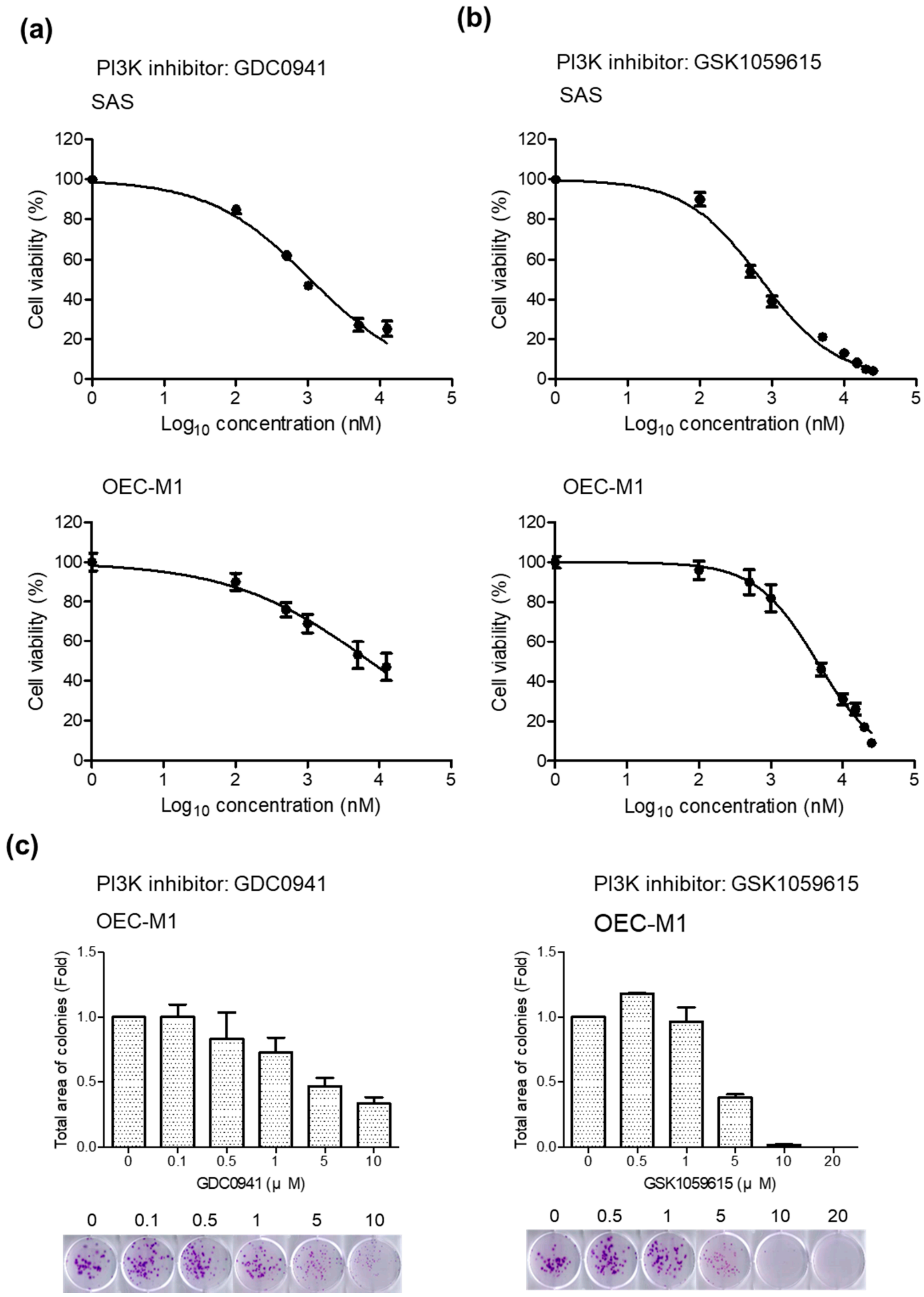

Figure S10. Screening of PI3K inhibitors in OSCC cell lines. Dose-response curve showing the cytotoxic effect of the PI3K inhibitors GDC0941 (**a**) and GSK1059615 (**b**) in SAS and OEC-M1 cells, as assessed by MTT assays. Colony formation assays of OEC-M1 cells treated with GDC0941 or GSK1059615 at the indicated concentrations.

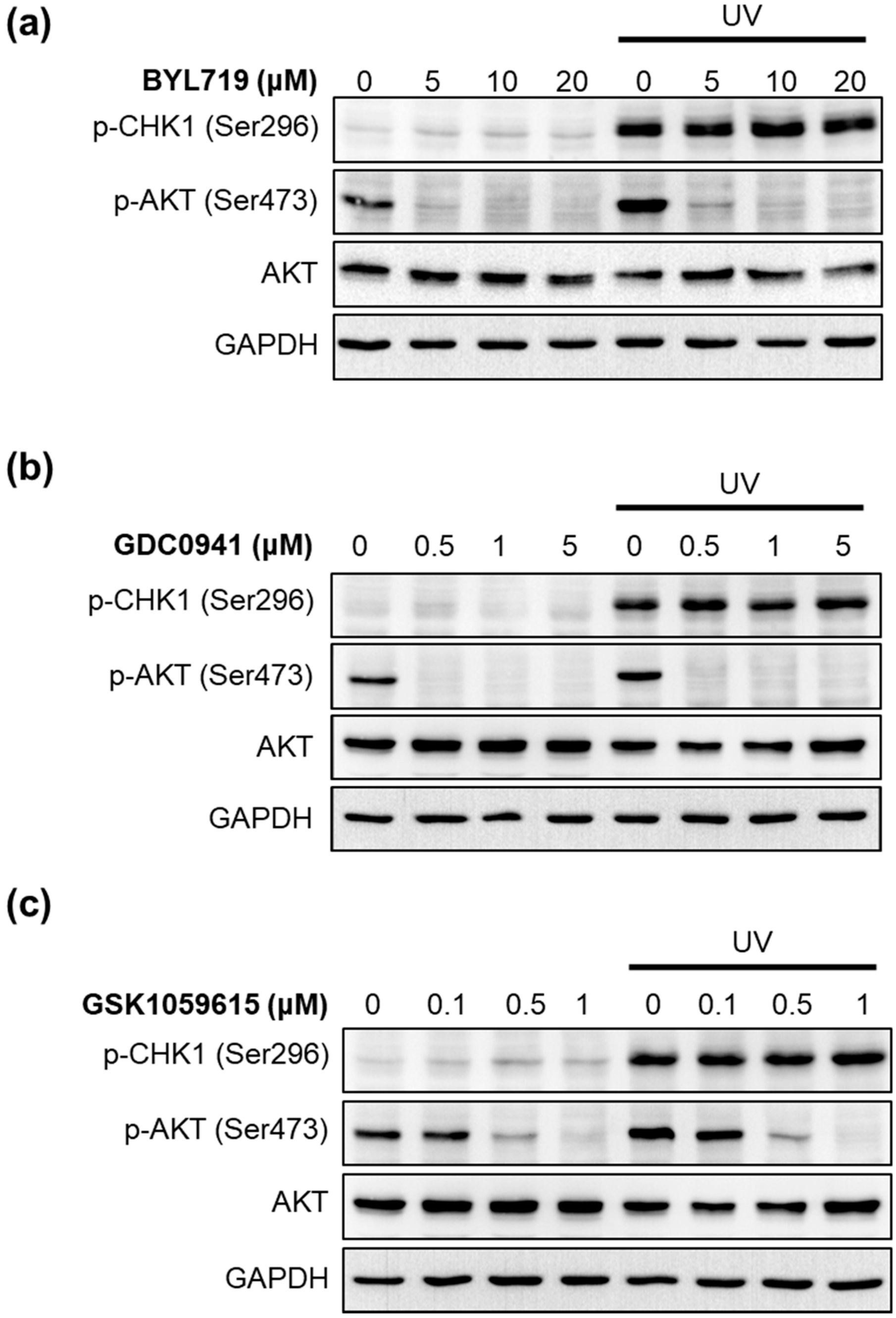

Figure S11. PI3K inhibitors effectively inhibit the phosphorylation of AKT. SAS cells seeded in 6-well plates were exposed to UV radiation (70 mJ/cm^2) for 2 h. Then, the cells were treated with DMSO only or with the PI3K inhibitors BYL719 (**a**), GDC0941 (**b**), and GSK1059615 (**c**) at the indicated doses for another 2 h. Cell lysates were collected, and Western blot analysis was performed to assess the levels of pCHK1 (Ser296), p-AKT (Ser4730) and total CHK1. GAPDH was used as the loading control.

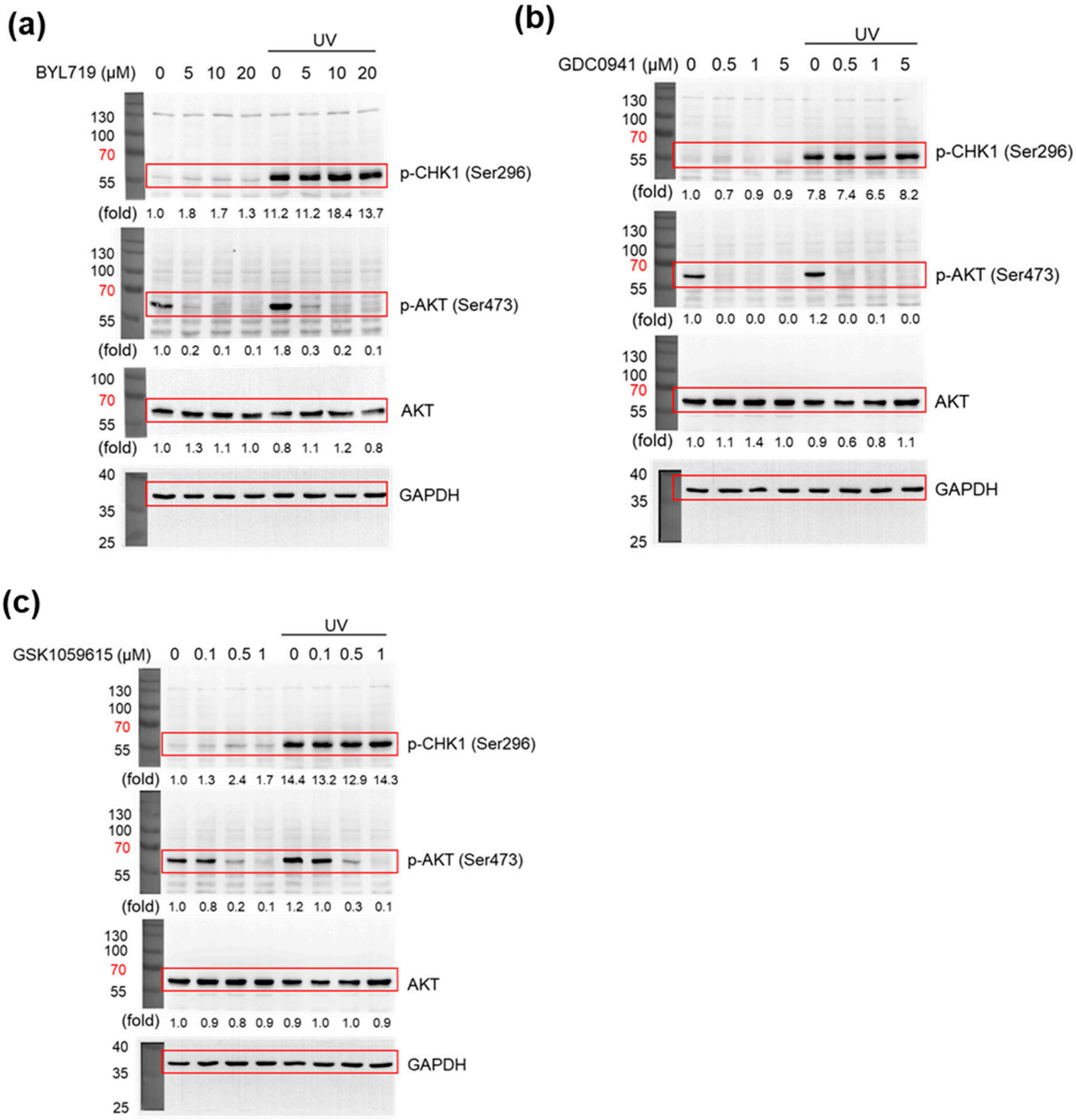

Figure S12. Uncropped scans of Western blots in Figure S11. In each lane, the band intensity ratios of p-CHK1, p-AKT, and AKT intensity to GAPDH were calculated. The ratios in the untreated sample were set to 1.

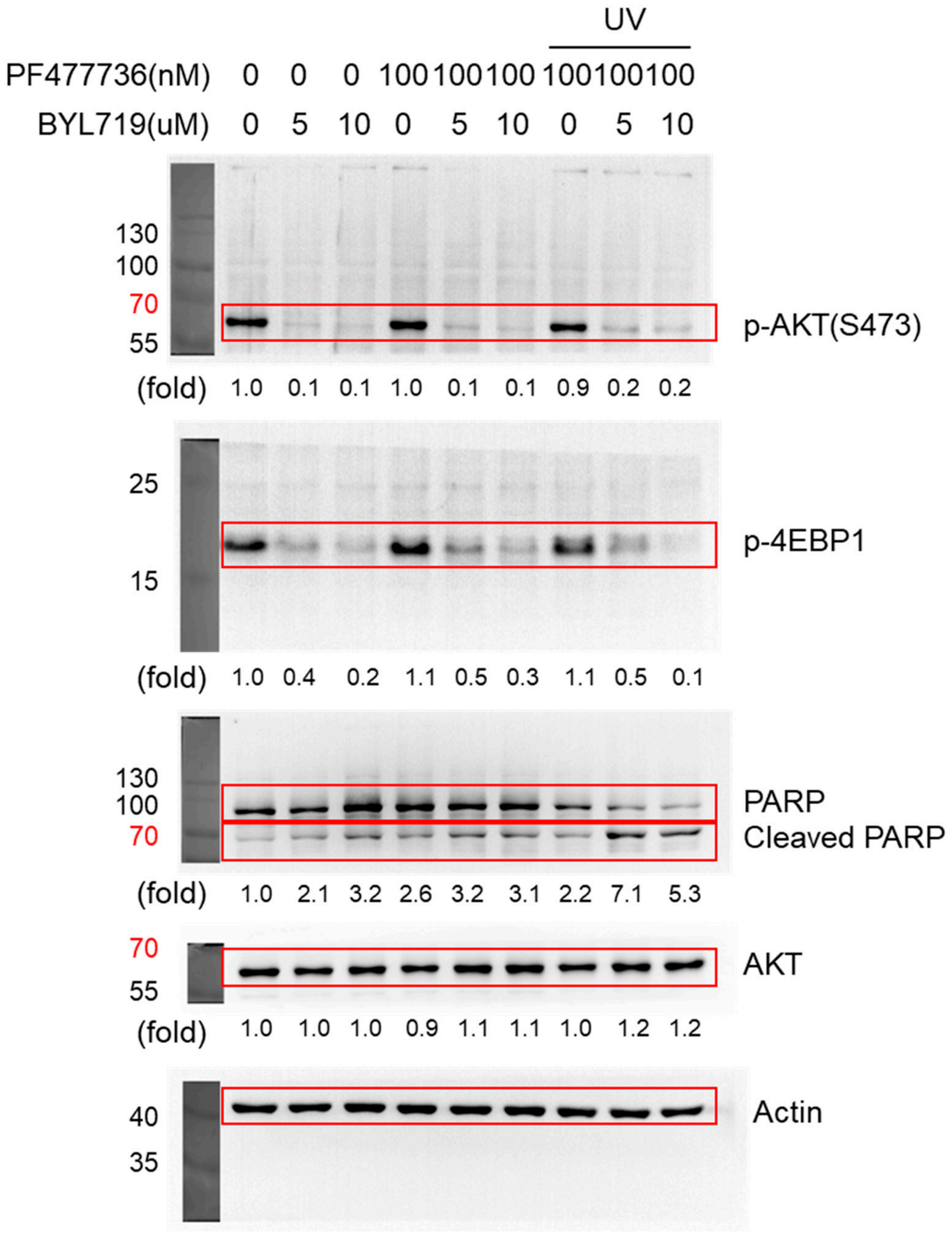

Figure S13. Uncropped scans of Western blots in Figure 4e. In each lane, the band intensity ratios of p-AKT, p-4EBP1, cleaved PARP, and AKT to Actin were calculated. The ratios in the untreated sample were set to 1.

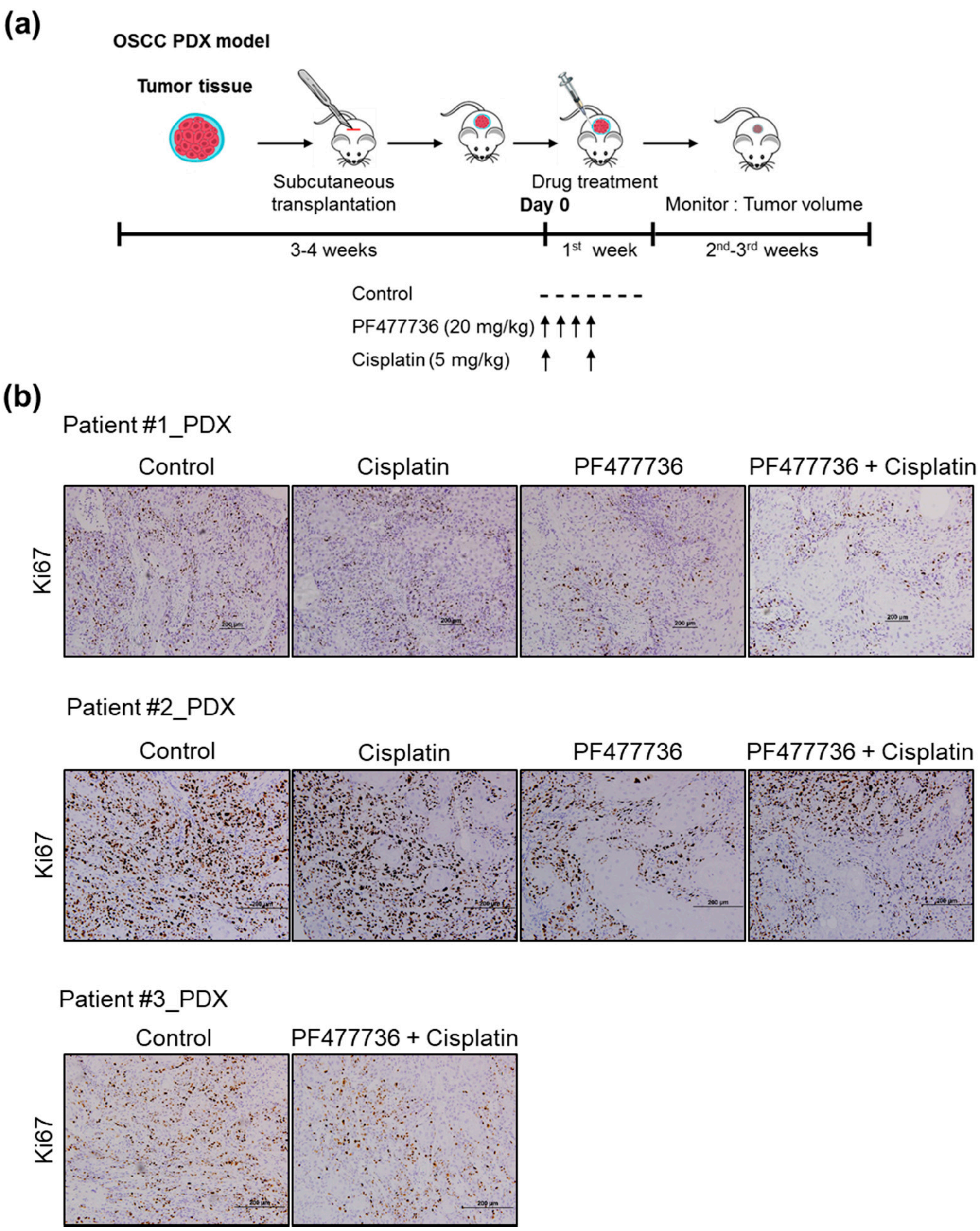

Figure 14. PF477736 and cisplatin treatment in OSCC PDX models**.** (**a**) The three OSCC PDX mouse models (patient #1 PDX, patient #2 PDX, patient #3 PDX) were established and grouped for different treatments, as follows: control, cisplatin only, CHK1 inhibitor PF477736 only, and PF477736 plus cisplatin. The treatment protocols and timelines are described in the Materials and Methods. (**b**) Representative Ki67 IHC staining images from OSCC PDXs. Ki67 staining was significantly reduced in cotreated tumors, suggesting a reduction in cancer cell proliferation.

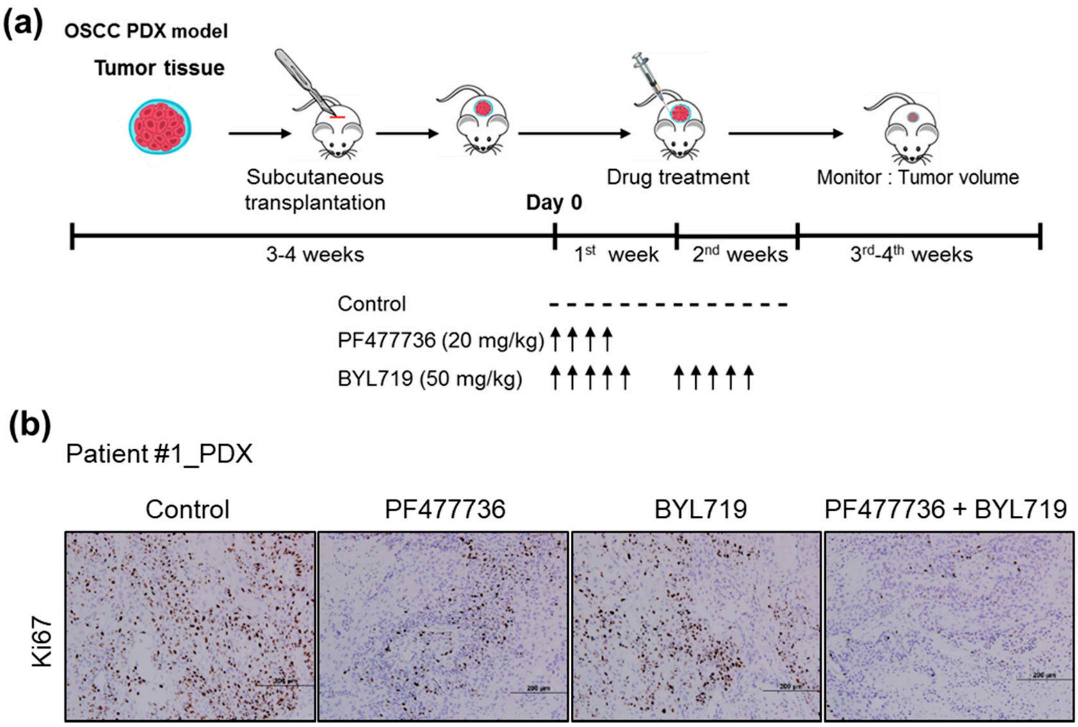

Figure S15. BYL719 and PF477736 treatment in OSCC PDX models. (**a**) Mice bearing OSCC PDXs were grouped for different treatments, as follows: control, CHK1 inhibitor PF477736 only, PI3K inhibitor BYL719 only, and PF477736 plus BYL719. The treatment protocols and timelines are described in the Materials and Methods. (**b**) Representative Ki67 IHC staining images from OSCC PDXs. Ki67 staining was significantly reduced in cotreated tumors, suggesting a reduction in cancer cell proliferation.

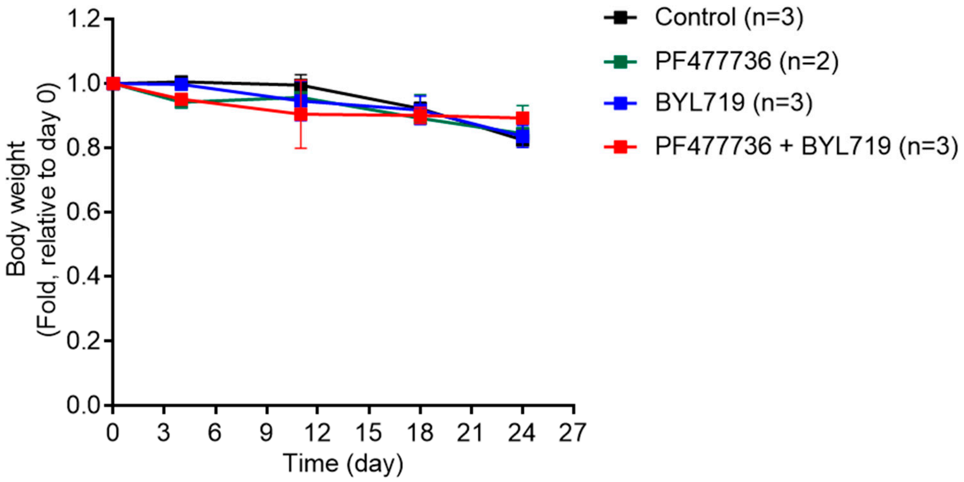

Figure S16. Body weight of OSCC PDX models after treatment. The PDX models were grouped as the control, PI3K inhibitor BYL719 only, CHK1 inhibitor PF477736 only, and PF477736 plus BYL719. Body weight was measured once a week.

© 2020 by the authors. Submitted for possible open access publication under the terms and conditions of the Creative Commons Attribution (CC BY) license (http://creativecommons.org/licenses/by/4.0/).
